# Mevalonate pathway inhibition reduces bladder cancer metastasis by modulating RhoB protein stability and integrin β1 localization

**DOI:** 10.1038/s42003-024-07067-8

**Published:** 2024-11-09

**Authors:** Gang Wang, Tianchen Peng, Liang Chen, Kangping Xiong, Lingao Ju, Kaiyu Qian, Yi Zhang, Yu Xiao, Xinghuan Wang

**Affiliations:** 1 Department of Urology, Laboratory of Precision Medicine, Zhongnan Hospital of Wuhan University, Wuhan, China; 2 Department of Biological Repositories, Human Genetic Resources Preservation Center of Hubei Province, Hubei Key Laboratory of Urological Diseases, Zhongnan Hospital of Wuhan University, Wuhan, China; 3 Euler Technology, ZGC Life Sciences Park, Beijing, China; 4 Center for Quantitative Biology, School of Life Sciences, Peking University, Beijing, China; 5 Medical Research Institute, Frontier Science Center for Immunology and Metabolism, Taikang Center for Life and Medical Sciences, Wuhan University, Wuhan, China; 6 Wuhan Research Center for Infectious Diseases and Cancer, Chinese Academy of Medical Sciences, Wuhan, China

**Keywords:** Bladder cancer, Prognostic markers, Cancer metabolism

## Abstract

The progression and outcome of bladder cancer (BLCA) are critically affected by the propensity of tumor metastasis. Our previous study revealed that activation of the mevalonate (MVA) pathway promoted migration of BLCA cells; however, the exact mechanism is unclear. Here we show that elevated expression of MVA pathway enzymes in BLCA cells, correlating with poorer patient prognosis by analyzing single-cell and bulk-transcriptomic datasets. Inhibition of the MVA pathway, either through knockdown of farnesyl diphosphate synthase (FDPS) or using inhibitors such as zoledronic acid or simvastatin, led to a marked reduction in BLCA cell migration. Notably, this effect was reversed by administering geranylgeranyl pyrophosphate (GGPP), not farnesyl pyrophosphate (FPP) or cholesterol, indicating the specificity of geranylgeranylation for cell motility. Moreover, we found that RhoB, a Rho GTPase family member, was identified as a key effector of the impact of the MVA pathway on BLCA metastasis. The post-translational modification of RhoB by GGPP-mediated geranylgeranylation influenced its protein stability through the ubiquitin-proteasome pathway. Additionally, overexpression of RhoB was found to block the membrane translocation of integrin β1 in BLCA cells. In summary, our findings underscore the role of the MVA pathway in BLCA metastasis, providing insights into potential therapeutic targets of this malignancy.

## Abstract (web summary)

RhoB plays an important role in the effect of MVA pathway inhibition on the migratory capacity of bladder cancer cells and its protein stability is closely related to GGPP-mediated geranylgeranyl modification.

## Introduction

Bladder cancer (BLCA) is the 10th most commonly diagnosed cancer worldwide, with more than 200,000 deaths annually^1^. Due to its recurring nature, BLCA requires long-term monitoring, increasing its economic burden^2^. Patients with metastatic BLCA have a poor prognosis, and lymph node-positive disease is an independent predictor of worse survival^3^. In addition, ~5% of metastatic cancer patients survive for at least 5 years postdiagnosis^4^. Thus, metastasis is an important factor contributing to the worsening prognosis of patients with BLCA and is one of the most important causes of death^5^. Therefore, studies on the molecular mechanisms underlying metastasis and the identification of novel targets or drugs are needed for the clinical management of BLCA.

Lipids are essential sources of energy during tumor development and metastasis and promote intercellular communication in the tumor microenvironment^6^. In a prospective cohort study, metabolic factors such as BMI, cholesterol, and triglycerides were found to be positively associated with BLCA risk^7^. Cholesterol, an important class of lipids, is involved in the regulation of the fluidity and permeability of lipid bilayers and is also an important component of lipid rafts^8^. In BLCA, compared to that in RT4 cells (noninvasive BLCA cells), the cholesterol content in T24 cells (high-grade invasive BLCA cells) is greater^9^. The mevalonate (MVA) pathway plays an important role in cholesterol synthesis and has been reported to be involved in the regulation of tumor metastasis^10,11^. Liu et al. discovered that activation of the MVA pathway promotes cholesterol biosynthesis and contributes to BLCA growth^12^. Based on our previous finding that simvastatin, an inhibitor of key enzymes of the MVA pathway, inhibits the proliferation and metastasis of BLCA cells, we confirmed that it affects the cell cycle distribution of BLCA cells through the PPARγ signaling pathway^13^. However, the mechanism through which the MVA pathway regulates BLCA cell metastasis is not yet clear.

Intermediate metabolites of the MVA pathway, such as farnesyl pyrophosphate (FPP) and geranylgeranyl pyrophosphate (GGPP), act as substrates for protein isoprenylation and are involved in the post-translational modification of small GTPases, such as Ras and Rho family GTPases, which are essential for the invasion and metastasis of a variety of cancers^14–16^. Farnesyl diphosphate synthase (FDPS) is an enzyme of the MVA pathway that catalyzes the synthesis of FPP and GGPP from isopentenyl pyrophosphate (IPP) and dimethylallyl pyrophosphate (DMAPP)^17^. It is also the target of nitrogen-containing bisphosphonates (N-BPs), a class of bone antiresorptive drugs used to treat osteoporosis and metastatic bone disease^18^. A study in patients with bone metastases from BLCA showed that zoledronic acid (ZOL), an N-BPs, improved overall survival compared to the effect of the placebo^19^. In addition, several studies have reported that ZOL inhibits the proliferation of BLCA cells^20,21^. However, whether the effect of ZOL on BLCA is associated with mevalonate pathway inhibition and the mechanism by which ZOL improves the prognosis of patients with metastatic BLCA need to be further investigated.

In this study, we investigated the impact of inhibiting the MVA pathway on the metastatic potential of BLCA and the underlying mechanisms involved. We discovered that FDPS, an enzyme whose expression is elevated in BLCA, is governed by the PSME3-mediated, ubiquitin-independent proteasome system. Intriguingly, while the migratory inhibition caused by targeting FDPS or using inhibitors such as ZOL or simvastatin was counteracted by GGPP, FPP and cholesterol did not produce the same effect. Further investigations revealed that the protein RhoB, a member of the Rho GTPases, is a critical effector of MVA pathway inhibition on BLCA cell migration. The stabilization of the RhoB protein appears to be modulated by GGPP through the ubiquitin-proteasome pathway. These findings highlight the complex role of the MVA pathway and RhoB in BLCA metastasis and suggest potential targets for therapeutic intervention.

## Results

### Genetic alterations of MVA pathway-related enzymes across urologic tumors

Based on the findings of previous studies^22^, the following enzymatic components of the MVA pathway were identified as MVA pathway**-**related enzymes (MREs): ACAT1 (acetyl-CoA acetyltransferase 1), ACAT2 (acetyl-CoA acetyltransferase 2), FDFT1 (farnesyl-diphosphate farnesyltransferase 1), FDPS (farnesyl diphosphate synthase), GGPS1 (geranylgeranyl diphosphate synthase 1), HMGCL (3-hydroxy-3-methylglutaryl-CoA lyase), HMGCR (hydroxy-3-methylglutaryl-CoA reductase), HMGCS1 (3-hydroxy-3-methylglutaryl-CoA synthase 1), IDI1 (isopentenyl-diphosphate delta isomerase 1), IDI2 (isopentenyl-diphosphate delta isomerase 2), MVD (MVA diphosphate decarboxylase), MVK (MVA kinase) and PMVK (phosphomevalonate kinase). In our study, the genomic data of five urologic tumor types comprising BLCA, kidney chromophobe (KICH), kidney renal clear cell carcinoma (KIRC), kidney renal papillary cell carcinoma (KIRP) and prostate adenocarcinoma (PRAD) from the TCGA dataset, including genetic variation, somatic copy number alteration (SCNA), and mRNA expression data, were analyzed to discern MREs dysregulation patterns.

**Fig. 1:**
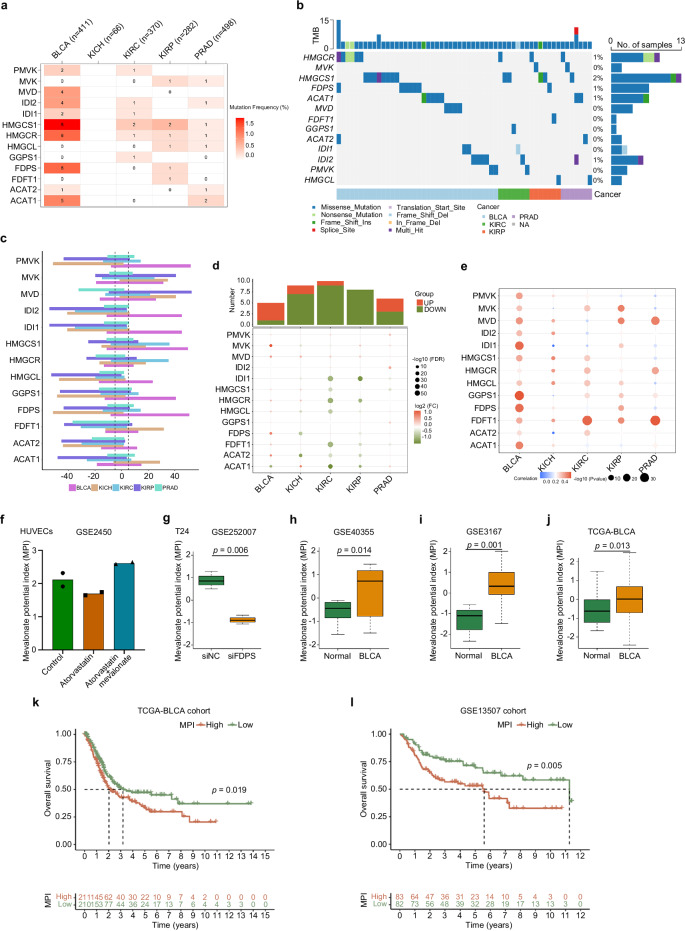
Genetic alterations of MVA pathway-related enzymes across urologic tumors. **a** Mutation frequency of MREs across cancers in TCGA database. The *X*-axis represents each cancer, and the *Y*-axis represents the frequency of mutations in MREs. The data were downloaded from the Xena Browser (https://xenabrowser.net/). **b** Mutation types, mutation frequencies and tumor mutation burden (TMB) of MREs across cancers. The *X*-axis represents each cancer, the *Y*-axis represents the different MREs and the number of samples with mutations, and the top is the TMB. **c** Histogram shows the frequency of SCNAs for each MRE in each cancer type. **d** The gene expression patterns of MREs across cancers. The *X*-axis represents each cancer, the *Y*-axis represents the different MREs, and the top represents the statistics of the number of upregulated and downregulated MREs in different types of tumors. **e** The Spearman’s correlation between somatic copy number alterations and the expression of MREs. MPIs were validated in multiple datasets: GSE2450 (**f**), GSE252007 (**g**), GSE40355 (**h**), GSE3167 (**i**), and TCGA-BLCA (**j**). Survival analysis of BLCA patients with high MPI or low MPI from the TCGA dataset (**k**) and GSE13507 dataset (**l**). Statistical significance was ascertained by two-tailed unpaired Student’s *t*-tests (**g**–**j**) and the log-rank test of Kaplan–Meier analysis (**k** and **l**). The data are shown as the means ± SD. BLCA bladder urothelial carcinoma, KICH kidney chromophobe, KIRC kidney renal clear cell carcinoma, KIRP kidney renal papillary cell carcinoma, PRAD prostate adenocarcinoma.

The frequency of nonsynonymous mutations was greater in BLCA than in other urologic tumors (Fig. 1a). Additionally, compared to other MREs, HMGCR and HMGCS1 had relatively high mutation frequencies in numerous urologic tumors, including BLCA, KIRC, KIRP and PRAD (Fig. 1b). The percentages of SCNA were analyzed and found that SCNA occurred at high rates (over 5% of all samples) in the BLCA, however, in KICH and KIRP, most MREs had a lower frequency of SCNA (Fig. 1c).

Beyond genetic alterations, we examined the gene expression patterns of MREs in tumors and normal tissues for every cancer type. There was differential expression of each MRE in at least one type of cancer. There were differences in MREs expression patterns among different urinary tumors. In BLCA, most MREs were upregulated, while in KICH, KIRC and KIRP, most were downregulated (Fig. 1d). SCNA in tumors is closely associated with the regulation of gene expression, so we further assessed the effect of SCNA on gene expression in MREs. The results showed that the expression of most MREs was associated with SCNA (Fig. 1e). The above results based on the analysis of the TCGA dataset showed that genetic or expression alterations of MREs are specific to BLCA compared to other urologic tumors and deserve further investigation.

To further reveal the potential role of MREs in regulating tumorigenesis, we analyzed BLCA single-cell sequencing results from public databases (GSE190888). A total of 36,424 filtered cells in the dataset were subjected to bioinformatics analysis, and clustering analysis identified 11 cell clusters (Supplementary Fig. 1a). Further analysis of the canonical cell type-specific markers revealed seven classical cell types, namely, basal tumor cells, urothelial cells, endothelial cells, T cells, macrophages, muscle cells and fibroblasts (Supplementary Fig. 1b). The MREs ACAT1, ACAT2, FDFT1, FDPS, HMGCL and PMVK were highly expressed in basal tumor cells and urothelial cells (Supplementary Fig. 1c–h).

### MVA pathway activation in BLCA and indicates a worse prognosis

To explore the role of the MVA pathway in tumorigenesis and identify factors or biological processes associated with this pathway, we calculated the MVA potential index (MPI) through ssGSEA using the enrichment score of core machine components. In this study, we calculated the MPI using an independent GEO dataset (GSE2450) of HUVECs treated with atorvastatin, a drug reported to be an inhibitor of MVA synthesis^23^. The MPI was significantly decreased by atorvastatin, but the addition of MVA reversed the MPI (Fig. 1f). In addition, we calculated the MPI of BLCA T24 cells transfected with siRNA targeting *FDPS*, a key enzyme of the MVA pathway^17^. As shown in Fig. 1g, *FDPS* knockdown clearly decreased the MPI compared to that of the controls. Since the inhibition of the MVA pathway by atorvastatin treatment or *FDPS* knockdown was unequivocal, analyses based on the validation of the above two independent datasets, revealed that the MPI could be used to represent the potential level of MVA pathway activity based on transcriptomic data. Furthermore, we found that the MPI differed between tumor tissues and normal tissues when using independent BLCA gene expression datasets. The MPI was significantly greater in BLCA samples than in normal samples, as shown in Fig. 1h–j. Therefore, we concluded that the MVA pathway was activated in BLCA tissues.

To further understand the clinical relevance of MVA pathway activation in cancer, we investigated the role of MPI in the survival of patients with BLCA. Our results showed that BLCA patients with high MPIs had worse overall survival in both the TCGA-BLAC cohort (Fig. 1k) and the GSE13507 cohort (Fig. 1l), suggesting that MPI is a risk factor for BLCA. As a result, further research into the functional roles of the MVA pathway in cancer progression is warranted.

### FDPS is highly expressed in BLCA and regulated by PSME3 for protein stability

**Fig. 2:**
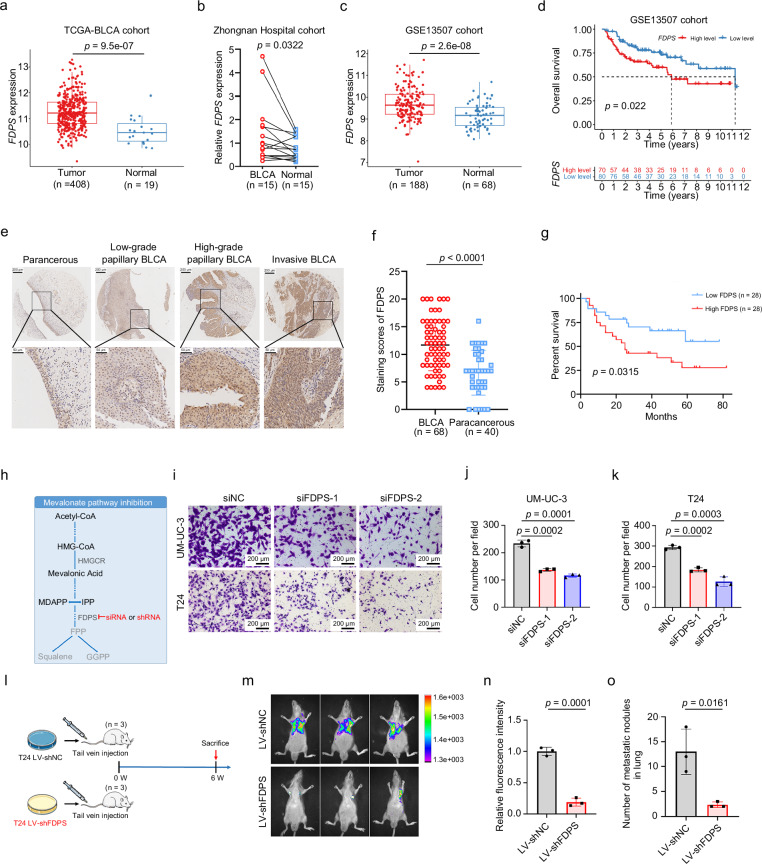
FDPS is upregulated in BLCA and promotes BLCA cell proliferation and metastasis. **a** The mRNA level of *FDPS* in BLCA (*n* = 408) and normal tissues (*n* = 19) in TCGA-BLCA (RNA-seq data). **b** The mRNA levels of *FDPS* in BLCA (*n* = 15) and paracancerous tissues (*n* = 15) in the Zhongnan Hospital cohort were measured by qRT-PCR. **c** The mRNA expression level of *FDPS* in BLCA (*n* = 188) and normal tissues (*n* = 68) in the GSE13507 cohort (RNA-seq data). **d** OS analysis of patients with BLCA who had different *FDPS* mRNA levels in the GSE13507 dataset. **e** Representative images of IHC staining analysis of FDPS protein in BLCA and paracancerous tissues from tissue microarray. The scale bars are 200 and 50 μm. **f** A statistical graph of staining scores of FDPS expression in BLCA (*n* = 68) and paracancerous tissues (*n* = 40). **g** The overall survival of patients with different FDPS protein levels in tissue microarray. The patients were divided into a high FDPS protein level group (*n* = 28) and a low FDPS protein level group (*n* = 28) according to the median staining scores of FDPS expression. The patients with missing survival data were not included. **h** Schematic representation of MVA pathway inhibition by targeting FDPS with siRNA or shRNA. Representative images (**i**) and statistical analysis of transwell migration assays results for the indicated groups of UM-UC-3 cells (**j**) and T24 cells (**k**) with FDPS knockdown (*n* = 3). The scale bar is 200 μm. **l** Schematic representation of the mouse pulmonary metastasis model constructed using LV-T24-shNC or LV-T24-shFDPS cells. Images of lung fluorescence after T24-shNC or T24-shFDPS cells were injected into the tail veins of BALB/C-nude mice for 6 weeks (**m**), and the fluorescence intensity of the lung metastases was quantified (*n* = 3) (**n**). **o** Statistical analysis of the number of metastatic nodules in H&E-stained mouse lung tissue sections (*n* = 3). The *n* number represents *n* biologically independent experiments in each group. Statistical significance was ascertained by two-tailed paired Student’s *t*-tests (**b**), two-tailed unpaired Student’s *t*-tests (**a**, **c**, **f**, **n**, and **o**) and one-way ANOVA with Dunnett’s multiple comparisons test (**j** and **k**) and the log-rank test of Kaplan–Meier analysis (**d** and **g**). The data are shown as the means ± SD.

Our previous study revealed that targeted inhibition of HMGCR, a key enzyme of the MVA pathway, attenuated the proliferation and metastasis of BLCA cells^13^. FDPS is a downstream enzyme of HMGCR in the MVA pathway; however, its expression and function in BLCA have not been described. Therefore, we analyzed the expression of FDPS in several publicly available BLCA datasets and in collections of BLCA samples. The results showed significantly elevated *FDPS* mRNA expression in BLCA tissues compared to paracancerous or normal tissues, as evident in the TCGA-BLCA dataset (Fig. 2a), the Zhongnan Hospital cohort (Fig. 2b), and the GSE13507 cohort (Fig. 2c). Additionally, *FDPS* was significantly positively correlated with lymph node metastasis in the TCGA-BLCA cohort (Supplementary Fig. 2a) and with tumor T stage in the GSE32548 cohort (Supplementary Fig. 2b). Meanwhile, we determined that BLCA patients with higher *FDPS* expression in both the GSE13507 cohort (Fig. 2d) and the GSE32548 cohort (Supplementary Fig. 2c) had poorer overall survival. Furthermore, by analyzing the tissue microarray (containing 68 BLCA specimens and 40 paracancerous tissues) (Fig. 2e–g), we found that FDPS protein levels were upregulated in BLCA tissues compared to paracancerous tissues (Fig. 2f), and patients with higher FDPS protein levels had poorer overall survival (Fig. 2g). The above results suggest that FDPS may play a role in BLCA tumorigenesis and progression.

Previous studies have primarily explored the reasons for the high expression of FDPS in tumors at the transcriptional level^24^, but rarely at the post-translational modification level. Therefore, in this study, we explored potential FDPS-associated proteins by IP–MS, and the top-ranked protein, PSME3, which is an important activator of the 20S proteasome and regulates the degradation of proteins, was of interest (Supplementary Fig. 3a). Co-IP analysis further confirmed the interaction between FDPS and PSME3 (Supplementary Fig. 3b). Moreover, we found colocalization of Flag-FDPS and HA-PSME3 in T24 and UM-UC-3 cells by immunofluorescence staining (Supplementary Fig. 3c). After T24 and UM-UC-3 cells were transfected with the HA-PSME3 plasmid, FDPS protein was reduced (Supplementary Fig. 3d). FDPS protein levels were decreased by PSME3 in a dose-dependent manner, which was blocked by the addition of the proteasome inhibitor MG132 (Supplementary Fig. 3e). To further determine whether PSME3 affects the protein stability of FDPS, a cycloheximide (CHX) assay was performed, which revealed that PSME3 overexpression accelerated the degradation of FDPS (Supplementary Fig. 3f-g). We also observed that PSME3 did not affect FDPS polyubiquitination (Supplementary Fig. 3f–h). Accordingly, we suggest that FDPS protein stability is regulated by the PSME3-mediated ubiquitin-independent proteasome system, which is consistent with its previously reported function^25^. In addition, overexpression of PSME3 led to reduced migration in BLCA cells, whereas co-overexpression of FDPS and PSEM3 reversed the migratory inhibition induced by PSME3 overexpression (Supplementary Fig. 3i–k).

### MVA pathway inhibition by FDPS knockdown affects the proliferation and metastasis of BLCA cells

We explored the effect of MVA pathway inhibition on the BLCA phenotype by transfecting two FDPS siRNAs to knock down *FDPS* in BLCA cells (Fig. 2h). The knockdown efficiency of two FDPS siRNAs in BLCA cells was confirmed by qRT-PCR (Supplementary Fig. 4a) and Western blotting (Supplementary Fig. 4b). The cell viability determined by MTT (Supplementary Fig. 5a-c) and clonogenic survival (Supplementary Fig. 5d–f) assays suggested that the proliferation of BLCA cells was significantly inhibited after *FDPS* knockdown. A transwell migration assay was used to measure the migratory ability of BLCA cells, and the results showed that *FDPS* knockdown significantly inhibited the migration of BLCA cells (Fig. 2i–k and Supplementary Fig. 5g, h).

To further explore the effect of FDPS on BLCA metastasis in vivo, we established a mouse pulmonary metastasis model using T24 cells with stable *FDPS* knockdown (Fig. 2i). The knockdown efficiency of LV-shNC and LV-shFDPS in BLCA cells was confirmed by qRT-PCR (Supplementary Fig. 5i) and western blotting (Supplementary Fig. 5j). After 6 weeks of tail vein injection, the fluorescence intensity in the lungs of the LV-shFDPS group mice (*n* = 3) was relatively lower than that in the LV-shNC group (*n* = 3) (Fig. 2m, n). Moreover, H&E staining of lung tissues from the two groups of mice showed that the size and number of lung metastatic nodules were reduced in the LV-shFDPS group (Fig. 2o and Supplementary Fig. 5k).

### ZOL-mediated inhibition of the MVA pathway affects the proliferation and metastasis of BLCA cells

**Fig. 3:**
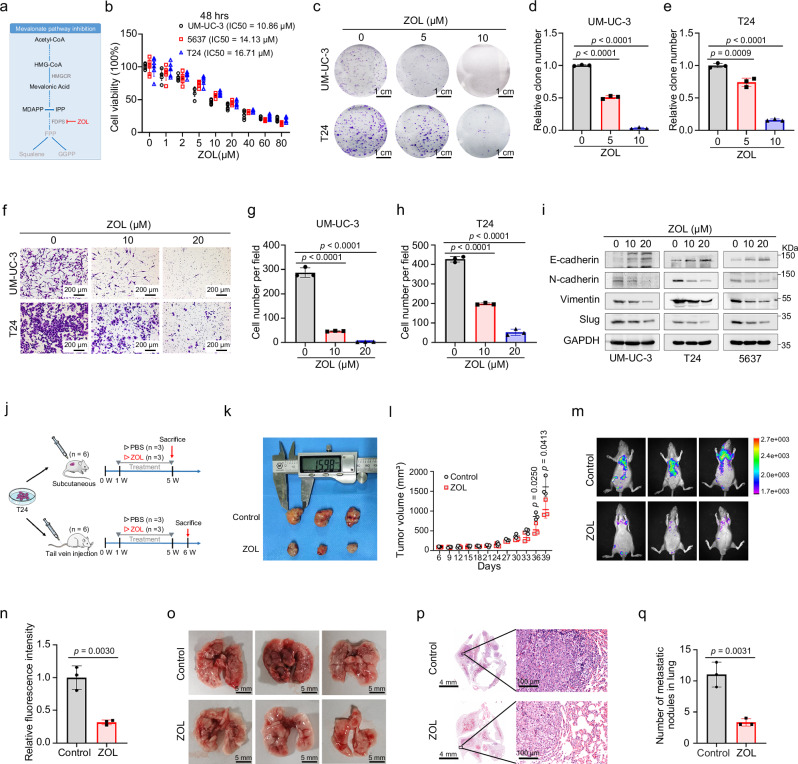
MVA pathway inhibition by ZOL affects the proliferation and metastasis of BLCA cells in vivo and in vitro. **a** Schematic representation of MVA pathway inhibition by ZOL. **b** An MTT assay was performed to detect changes in the proliferation of BLCA cells (UM-UC-3, T24 and 5637) after treatment with different concentrations (0, 1, 2, 5, 10, 20, 40, 60 and 80 μM) of ZOL for 48 h (*n* = 6). Representative images (**c**) and statistical analysis (**d** and **e**) of colony formation assays from the indicated groups after treatment with ZOL at different concentrations (0, 5 and 10 μM) in UM-UC-3 cells (**d**) and T24 cells (**e**) for 48 h (*n* = 3). The scale bar is 1 cm. Representative images (**f**) and statistical graph (**g** and **h**) of transwell assays from the indicated groups after treatment with ZOL at different concentrations (0, 10 and 20 μM) in UM-UC-3 cells (**g**) and T24 cells (**h**) for 48 h (*n* = 3). The scale bar is 200 μm. **i** Western blot analyses of EMT-related proteins in ZOL-treated UM-UC-3, T24 and 5637 cells. GAPDH was used as the loading control. **j** Schematic representation of the effects of ZOL-mediated inhibition of the MVA pathway on BLCA proliferation and metastasis in a mouse xenograft model and pulmonary metastasis model. **k** Gross view of a subcutaneous tumor; the upper side represents the control group (*n* = 3), while the lower side represents the ZOL treatment group (*n* = 3). **l** Tumor volume was calculated during the experiment. Images of lung fluorescence in the control group and ZOL treatment group (**m**) and quantification of the fluorescence intensity of lung metastases (*n* = 3) (**n**). Images of dissected whole lungs (**o**) and representative images of H&E-stained mouse lung tissue sections (*n* = 3) (**p**); the scale bars are 4 mm and 100 μm. Statistical analysis of the number of metastatic nodules in H&E-stained mouse lung tissue sections (**q**). The *n* number represents *n* biologically independent experiments in each group. Statistical significance was ascertained by two-tailed unpaired Student’s *t*-tests (**l**, **n** and **q**) and one-way ANOVA with Dunnett’s multiple comparisons test (**d**, **e**, **g**, and **h**). The data are shown as the mean ± SD.

Subsequently, ZOL, an FDPS inhibitor, was used to further explore the effects of MVA pathway inhibition on BLCA cells (Fig. 3a). BLCA cells UM-UC-3, 5637 and T24 were treated with ZOL at different concentrations (0, 1, 2, 5, 10, 20, 40, 60 and 80 μM) for 24 h (Supplementary Fig. 6a), 48 h (Fig. 3b) or 72 h (Supplementary Fig. 6b). The cell viability determined by the MTT assay suggested that the proliferation of UM-UC-3 (the IC50 values at 48 and 72 h were 10.86 and 0.9406 μM, respectively), 5637 (the IC50 values at 48 and 72 h were 14.13 and 4.380 μM, respectively) and T24 cells (the IC50 values at 48 and 72 h were 16.71 and 1.866 μM, respectively) were significantly inhibited by ZOL treatment in a time- and dose-dependent manner. By analyzing the IC50 values of the three BLCA cell lines (UM-UC-3, 5637 and T24) treated with different concentrations of ZOL in the above results, and combining the ZOL concentrations reported in previous studies^26,27^, we selected 10 and 20 μM ZOL-treated BLCA cells for our subsequent experiments.

The clonogenic survival assay showed that, compared to that in the control group, the colony formation efficiency of ZOL-treated BLCA cells was significantly inhibited (Fig. 3c–e and Supplementary Fig. 6c, d). When we performed the clonogenic survival assay after treating UM-UC-3 cells with 10 μM ZOL, it was already difficult for the cells to form colonies, so 5 and 10 μM ZOL were chosen for the treatment of BLCA cells in this study.

To assess the impact of ZOL on BLCA cell metastasis, transwell migration and wound healing assays were employed. After 24 h of treatment with different ZOL concentrations (0, 10 and 20 μM), the migration was significantly reduced in all ZOL-treated BLCA cells (UM-UC-3, 5637 and T24) (Fig. 3f–h and Supplementary Fig. 6e, f). Similar results were replicated by wound healing assay (Supplementary Fig. 7), reconfirming the significant decrease in migration caused by ZOL treatment. Western blotting was used to analyze the changes in proteins involved in the epithelial–mesenchymal transition (EMT) process, revealing upregulation of E-cadherin and downregulation of N-cadherin, Vimentin and Slug in BLCA cells after ZOL treatment (Fig. 3i).

To explore the in vivo effect of ZOL on BLCA cell proliferation and metastasis, xenograft and pulmonary metastasis models were established (Fig. 3j). In the xenograft model, compared with that in the control group (*n* = 3), the tumor growth in the ZOL group (*n* = 3) was significantly inhibited (Fig. 3k, l). Pulmonary metastasis models were established by tail vein injection of T24 cells, with mice divided into a control group (PBS injection) (*n* = 3) and a ZOL injection group (*n* = 3). As shown in Fig. 3m, n, ZOL treatment significantly suppressed in vivo migration compared to the control group. H&E staining of lung tissues showed that the size and number of lung metastatic nodules were reduced in the ZOL treatment group (Fig. 3o–q).

### GGPP restores migration inhibition of MVA pathway in BLCA

**Fig. 4:**
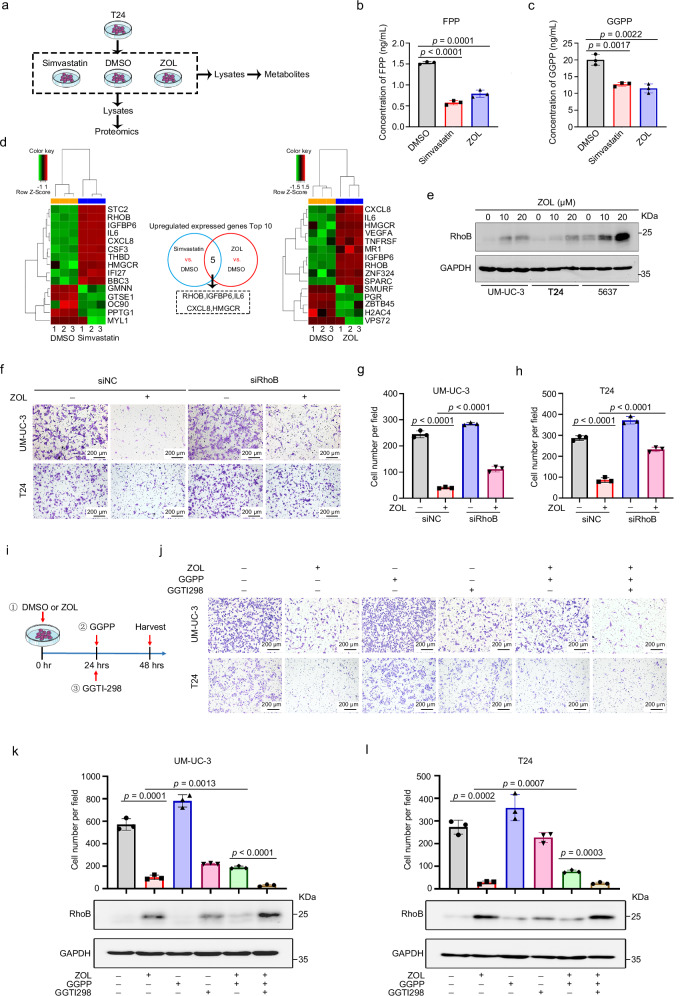
GGPP-mediated geranylgeranylation of RhoB protein is associated with the migration ability of BLCA cells. **a** Flowchart for the detection of MVA pathway intermediate metabolite (FPP and GGPP) levels and proteomic changes in BLCA cells after MVA pathway inhibition by ZOL or simvastatin. The contents of FPP (**b**) and GGPP (**c**) in T24 cells treated with ZOL (20 μM) (*n* = 3) or simvastatin (5 μM) (*n* = 3) were measured by LC–MS/MS analysis. **d** Heatmaps of proteins whose expression significantly changed in T24 cells after MVA pathway inhibition by ZOL or simvastatin, respectively. **e** Western blotting was performed to detect RhoB protein in BLCA cells after treatment with ZOL. GAPDH was used as the loading control. Representative images (**f**) and statistical graph (**g** and **h**) of transwell migration assays from the indicated groups after treatment of BLCA cells (UM-UC-3 and T24) with RhoB siRNA and ZOL (20 μM), respectively, or in combination (*n* = 3). The scale bar is 200 μm. **i** Schematic representation of BLCA cells treated with ZOL (20 μM), GGPP (5 μM) and GGTI298 (10 μM), alone or in combination. Representative images (**j**) and statistical analysis (**k** and **l**) of transwell migration assays from the indicated groups after treatment of BLCA cells (UM-UC-3 and T24) with ZOL (20 μM), GGPP (5 μM) and GGTI298 (10 μM), separately or in combination (*n* = 3). The scale bar is 200 μm. Changes in RhoB protein expression in different groups of UM-UC-3 (**k**) and T24 (**l**) cells were also detected by western blotting. GAPDH was used as the loading control. The *n* number represents *n* biologically independent experiments in each group. Statistical significance was ascertained by two-tailed unpaired Student’s *t*-test (**b**, **c**, **k** and **l**) and one-way ANOVA with Dunnett’s multiple comparisons test (**g** and **h**). The data are shown as the mean ± SD.

To investigate the mechanism of MVA pathway inhibition in BLCA, two MVA pathway inhibitors, simvastatin (an HMGCR inhibitor) and ZOL (an FDPS inhibitor), were applied to BLCA cells. The treated cells, along with control cells, underwent an MVA pathway intermediate metabolite assay and proteomic analysis (Fig. 4a). GGPP and FPP are important intermediate metabolites of the MVA pathway. Our results showed that the levels of FPP and GGPP were reduced in BLCA cells treated with simvastatin or ZOL (Fig. 4b, c).

To investigate whether FPP and GGPP are involved in the effect of BLCA on migration capacity through MVA pathway inhibition, FPP (Supplementary Fig. 8a–c) and GGPP (Supplementary Fig. 8d–f) were added to ZOL-treated BLCA cells. Transwell migration assays revealed that after incubating with GGPP, the ZOL-induced reduction in the migration rate was significantly attenuated, whereas the effect of FPP was not (Supplementary Fig. 8a–f). Consistent with the above findings, GGPP also reversed the suppressive effect of simvastatin on migration (Supplementary Fig. 8g–i) and *FDPS* knockdown (Supplementary Fig. 8j–l) in BLCA cells.

The MVA pathway is the central metabolic pathway for cholesterol biosynthesis, therefore, we also explored whether cholesterol is involved in the effect of BLCA on migration capacity through MVA pathway inhibition. Cholesterol was added to ZOL-, simvastatin-treated or *FDPS* knockdown BLCA cells, respectively. Transwell migration assays showed a slight increase in the migration of BLCA cells after the administration of cholesterol alone, however, cholesterol did not rescue the reduction in migration rate caused by ZOL-, simvastatin-treated or *FDPS* knockdown (Supplementary Fig. 9a–f). The total cholesterol content in simvastatin- or ZOL-treated BLCA cells was analyzed, and compared to that in control cells, the cholesterol content in the drug-treated BLCA cells was not significantly reduced (Supplementary Fig. 9g, h). Further qRT-PCR analysis revealed that the mRNA expression of *ABCA1* and *ABCG1*, which are responsible for the transport of cholesterol to the extracellular compartment^28^, were decreased, whereas the mRNA expression of *LDLR*, *NPC1L1* and *SCARB1*, which are responsible for the uptake of cholesterol^29^, were increased in simvastatin- or ZOL-treated cells (Supplementary Fig. 9i, j).

### Role of RhoB in attenuating BLCA metastasis induced by MVA pathway inhibition

In our investigation, BLCA cells were treated with simvastatin or ZOL, and subsequent proteomic analysis was performed on both treated and control cells. The heatmaps show proteins that were significantly upregulated (top 10) and proteins that were significantly downregulated (top 5) in T24 cells after simvastatin (Fig. 4d, left panel) and ZOL treatment (Fig. 4d, right panel), respectively. We examined the intersection of the significantly upregulated proteins (top 10) in the two groups and identified five proteins (Fig. 4d, middle panel), among which RhoB has been reported to be isoprenylated by GGPP or FPP and closely associated with tumor migration^30,31^.

Further validation confirmed that the significant upregulation of the RhoB protein following ZOL-mediated inhibition of the MVA pathway (Fig. 4e), simvastatin treatment (Supplementary Fig. 10a) or *FDPS* knockdown (Supplementary Fig. 10b) in BLCA cells. Importantly, we also found that MVA pathway inhibition did not increase *RhoB* mRNA expression (Supplementary Fig. 10c). Moreover, our results showed that the addition of GGPP to the culture media could reduce the upregulation of the RhoB protein induced by ZOL (Supplementary Fig. 10d), simvastatin (Supplementary Fig. 10e), or *FDPS* knockdown (Supplementary Fig. 10f).

To further investigate whether RhoB is involved in the MVA pathway-mediated regulation of the migratory ability of BLCA cells, we transfected RhoB siRNA into ZOL-treated BLCA cells or *FDPS* knockdown BLCA cells (Fig. 4f and Supplementary Fig. 10g–j). The transwell migration assay results revealed a significant attenuation of the inhibition of cell migration caused by ZOL (Fig. 4f–h) or *FDPS* knockdown (Supplementary Fig. 10h–j) upon RhoB siRNA transfection in T24 and UM-UC-3 cells.

### GGPP-mediated geranylgeranylation of RhoB protein associates with BLCA cell migration

GGTI298, a geranylgeranyl transferase inhibitor (GGTI), can inhibit the geranylgeranyl protein transferase (GGTase)-mediated geranylgeranyl acylation reaction. Moreover, RhoB proteins can also undergo geranylgeranylation. After the administration of ZOL, GGPP, or GGTI298 separately or in combination, changes in the migratory capacity of BLCA cells were examined via a transwell migration assay (Fig. 4i). The results showed that the effect of ZOL on the migration of BLCA cell lines (T24 and UM-UC-3) was restored by the addition of GGPP. However, this effect was subsequently reversed by the addition of GGTI298 (Fig. 4j–l). Western blot analysis revealed the RhoB protein levels were significantly higher in the ZOL, GGPP and GGTI298 combination groups compared to the ZOL and GGPP combination groups (Fig. 4j–l). The above results further confirmed that GGPP affects the protein levels of RhoB by mediating its geranylgeranylation, thus implicating it in the regulation of BLCA cell migration capacity via the MVA pathway.

### Geranylgeranylated RhoB protein susceptibility to ubiquitination-mediated degradation

**Fig. 5:**
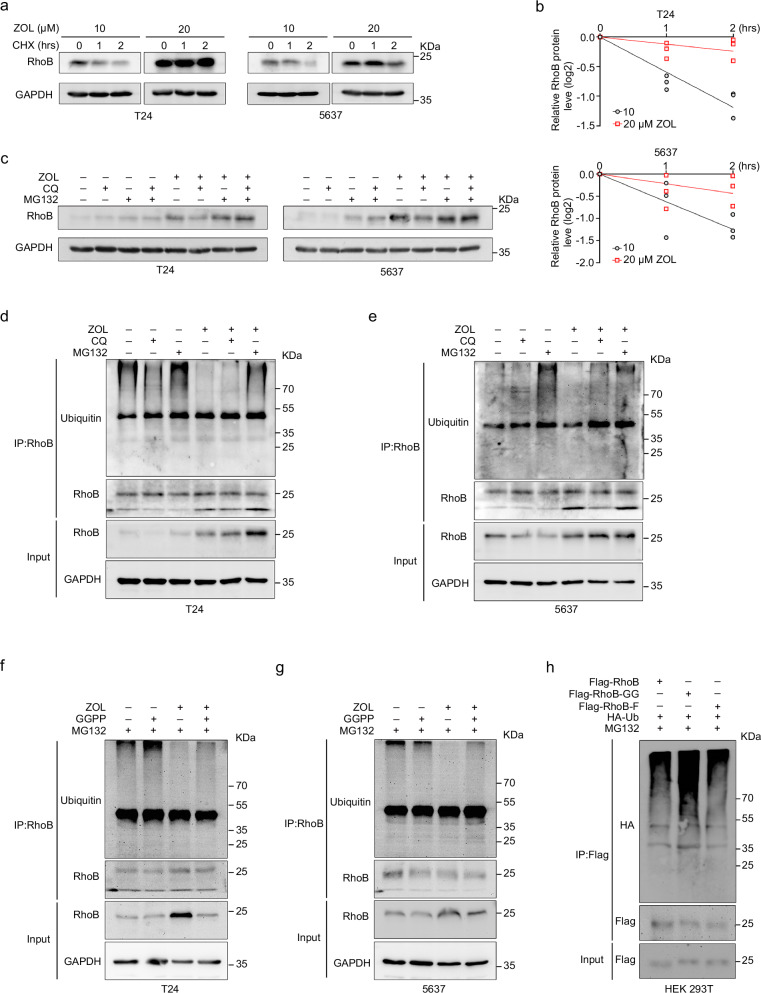
Geranylgeranylated RhoB protein is more susceptible to degradation through ubiquitination pathway. Western blot analysis of the effect of ZOL on RhoB degradation in BLCA cells (T24 and 5637) incubated with CHX (50 μg/mL) for the indicated durations (**a**) and statistical analysis (*n* = 3) (**b**). **c** BLCA cells (T24 and 5637) with or without ZOL were treated with CQ (50 μM) or MG132 (10 μM) for 8 h, after which RhoB protein expression was detected via western blotting. T24 (**d**) and 5637 (**e**) cells with or without ZOL were treated with CQ (50 μM) or MG132 (10 μM) for 8 h, and then ubiquitination experiments were performed to analyze the polyubiquitination of RhoB. T24 (**f**) and 5637 (**g**) cells with or without ZOL (20 μM) were treated with GGPP (5 μM) for 24 h, after which ubiquitination experiments were performed to analyze the polyubiquitination of RhoB. **h** 293 T cells were transfected with the described plasmids for 48 h and then treated with MG132 (10 μM) for 8 h, after which ubiquitination experiments were performed.

Since the RhoB protein in BLCA cells was significantly increased after MVA pathway inhibition, a CHX assay was performed to determine the half-life of RhoB protein degradation after ZOL administration at different concentrations (10 and 20 μM). The results showed that the degradation rate of the RhoB protein decreased significantly with increasing ZOL concentration in BLCA cells (Fig. 5a, b). To further determine the degradation pathway of the RhoB protein, we treated BLCA cells with ZOL, MG-132 (proteasome inhibitor) or chloroquine (CQ, lysosomal pathway inhibitor), alone or in combination. Western blot analysis revealed that the RhoB protein primarily underwent degradation through the ubiquitin-proteasome pathway (Fig. 5c). In vitro ubiquitination assays were then performed to assess the impact of ZOL on RhoB protein ubiquitination. Immunoprecipitation revealed that ZOL significantly decreased the polyubiquitination of RhoB protein, while MG-132 significantly increased the ubiquitination of the RhoB protein, but no effect was observed with CQ (Fig. 5d, e).

Considering our previous results indicating that GGPP could reverse the ZOL-induced increase in RhoB protein, we further examined changes in RhoB protein ubiquitination levels after treating BLCA cells separately or in combination with ZOL and GGPP. The results showed that GGPP could reverse the inhibitory effect of ZOL on RhoB protein ubiquitination (Fig. 5f, g). In addition, we found that RhoB-GG (geranylgeranylated-only RhoB) exhibited greater susceptibility to ubiquitination compared to RhoB (both geranylgeranylated and farnesylated RhoB) and RhoB-F (farnesylated-only RhoB) (Fig. 5h). Taken together, these results suggested that MVA pathway inhibition could inhibit the ubiquitination and degradation of the RhoB protein, which was associated with the depletion of GGPP in BLCA cells.

### RhoB inhibits BLCA cell proliferation and metastasis in vivo and in vitro

**Fig. 6:**
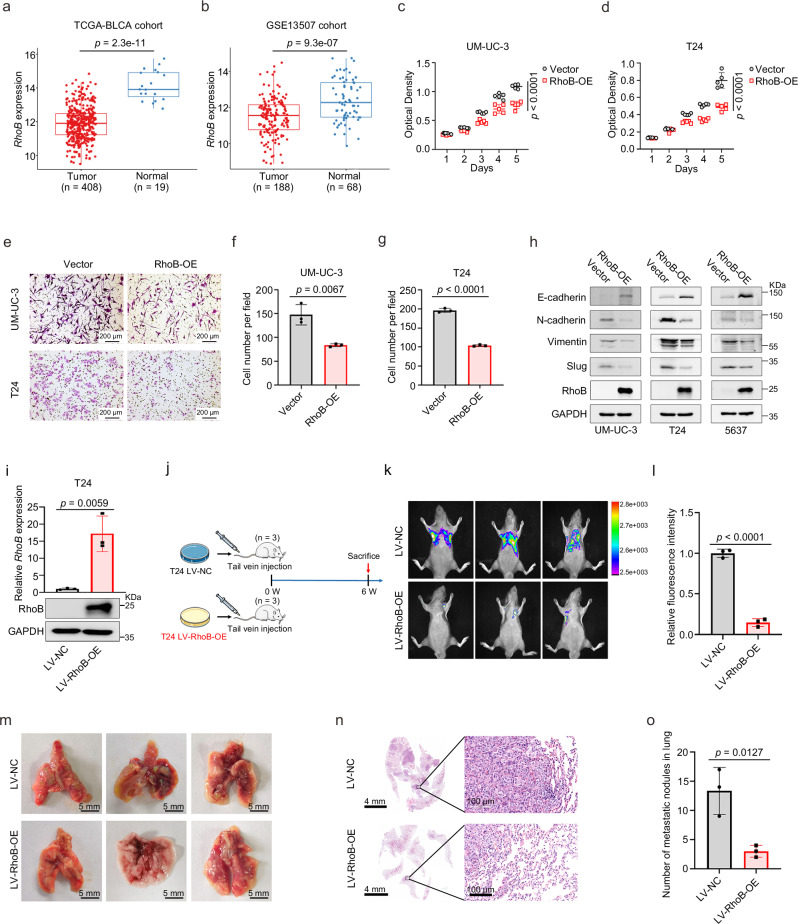
RhoB inhibits BLCA cell proliferation and metastasis. **a** The mRNA level of *RhoB* in BLCA (*n* = 408) and normal tissues (*n* = 19) in TCGA-BLCA (RNA-seq data). **b** The mRNA expression level of *RhoB* in BLCA (*n* = 188) and normal tissues (*n* = 68) in the GSE13507 cohort (RNA-seq data). An MTT assay was performed to detect changes in the proliferation of BLCA UM-UC-3 (**c**) and T24 (**d**) cells with or without RhoB overexpression (*n* = 5). Representative images (**e**) and statistical analysis (**f** and **g**) of colony formation assays of UM-UC-3 and T24 cells with or without RhoB overexpression (*n* = 3). The scale bar is 200 μm. **h** Western blot analyses of EMT-related proteins in UM-UC-3, T24 and 5637 cells with or without RhoB overexpression. GAPDH was used as the loading control. **i** Detection of RhoB expression at the mRNA and protein levels in LV-NC cells and LV-RhoB-OE cells by qRT-PCR and western blotting. **j** Schematic representation of the mouse pulmonary metastasis model constructed using T24 LV-NC cells and T24 LV-RhoB-OE cells. Images of lung fluorescence after T24 LV-NC cells and T24 LV-RhoB-OE cells were injected into the tail veins of BALB/C-nude mice for 6 weeks (**k**), and the fluorescence intensity of the lung metastases was quantified (*n* = 3) (**l**). Images of dissected whole lungs (**m**) and representative images of H&E-stained mouse lung tissue sections (**n**); the scale bars are 4 mm and 100 μm. **o** Statistical analysis of the number of metastatic nodules in H&E-stained mouse lung tissue sections (*n* = 3). The *n* number represents *n* biologically independent experiments in each group. Statistical significance was ascertained by two-tailed unpaired Student’s *t*-test (**a**–**d**, **f**, **g**, **i**, **l**, and **o**). The data are shown as the means ± SD.

RhoB protein expression was closely related to the migration ability of BLCA cells. To explore its clinical relevance in BLCA, we analyzed the expression level of *RhoB* in several publicly available BLCA datasets and found that *RhoB* expression was significantly reduced in BLCA (Fig. 6a, b and Supplementary Fig. 11a, b). To further study the functions of RhoB in BLCA, we constructed a RhoB overexpression plasmid to upregulate RhoB in BLCA cells. qRT-PCR was used to confirm the overexpression efficiency of the RhoB overexpression plasmid in BLCA cells (Supplementary Fig. 11c). Subsequently, MTT and transwell migration assays showed that the proliferation and migration of BLCA cells were significantly inhibited in cells transfected with the RhoB overexpression plasmid (Fig. 6c–g and Supplementary Fig. 11d–f). Western blotting of EMT-related proteins (E-cadherin, N-cadherin, Vimentin and Slug) revealed an upregulation of E-cadherin and a downregulation of N-cadherin, Vimentin and Slug in BLCA cells after RhoB overexpression (Fig. 6h), consistent with observations after ZOL treatment (Fig. 3i).

Pulmonary metastasis models were established using T24 cells stably overexpressing RhoB (LV-RhoB-OE) and control cells (LV-NC) to investigate the effect of RhoB on BLCA cell metastasis in vivo (Fig. 6i, j). After 6 weeks of injection, the fluorescence intensity of pulmonary metastatic tumors was measured to evaluate the migration capacity. The LV-RhoB-OE group (*n* = 3) exhibited relatively lower fluorescence intensity in lungs tissues compared to the LV-NC group (*n* = 3) (Fig. 6k, l). H&E staining of lung tissues showed that the size and number of lung metastatic nodules were reduced in the LV-RhoB-OE group (Fig. 6m–o).

### Impact of RhoB on integrin β1 translocation in BLCA cells

**Fig. 7:**
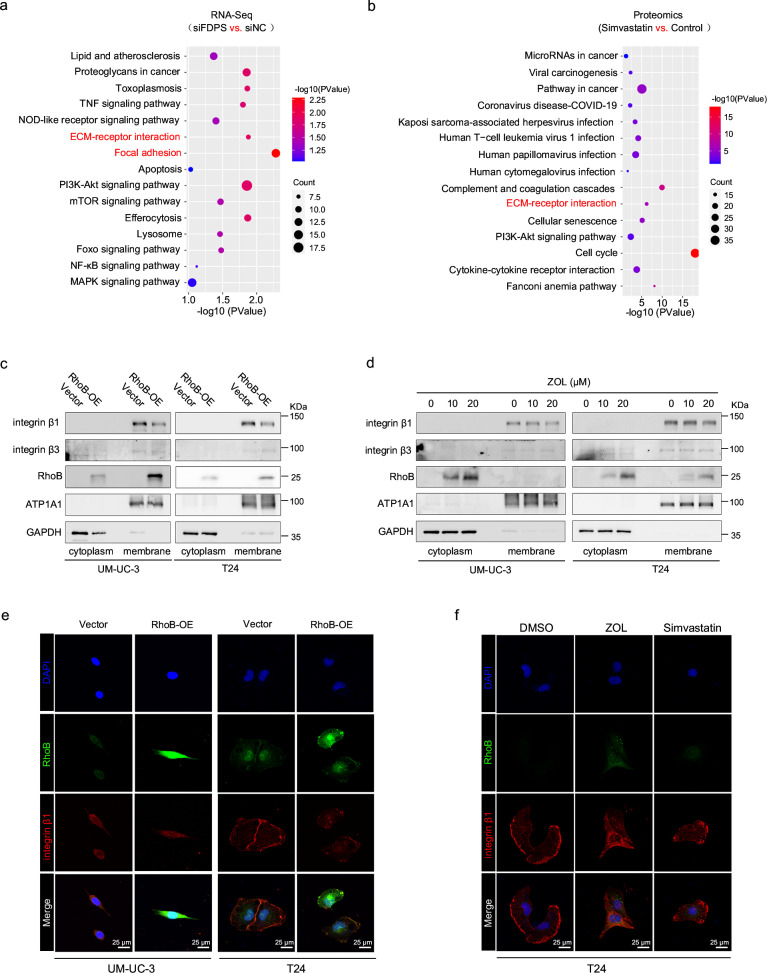
RhoB is involved in the translocation of integrin β1 from the cytoplasm to the membrane in BLCA cells. Pathway enrichment analysis of DEGs between *FDPS* knockdown T24 cells and siNC T24 cells (**a**) and differentially expressed proteins between simvastatin-treated T24 cells and control T24 cells (**b**). **c** The integrin β1 and β3 proteins in the cytoplasm and on the membrane of BLCA cells transfected with vector or RhoB plasmid were detected via western blotting. GAPDH and ATP1A1 were used as a loading control for the cytoplasmic and membrane proteins, respectively. **d** The integrin β1 and β3 proteins in the cytoplasm and on the membrane of BLCA cells treated with different concentrations of these agents (0, 10 and 20 μM) were detected via western blotting. GAPDH and ATP1A1 were used as a loading control for the cytoplasmic and membrane proteins, respectively. **e** The expression and localization of integrin β1 (red) or RhoB (green) in UM-UC-3 and T24 cells transfected with vector or RhoB plasmid were detected by immunofluorescence staining. Nuclei were stained with DAPI (blue). The scale bar is 25 μm. **f** The expression and localization of integrin β1 (red) and RhoB (green) in T24 cells treated with ZOL (20 μM) or simvastatin (5 μM) were detected via immunofluorescence staining. Nuclei were stained with DAPI (blue). The scale bar is 25 μm.

We performed KEGG pathway enrichment analysis of DEGs between *FDPS* knockdown and control T24 cells (Fig. 7a), as well as differentially expressed proteins between simvastatin-treated and control T24 cells (Fig. 7b), respectively, and found that both were enriched in pathways involving ECM-receptor interaction and focal adhesion (Fig. 7a, b). Furthermore, we performed RNA-seq analysis on RhoB overexpressed and control T24 cells. KEGG pathway enrichment analysis of DEGs revealed enrichment in pathways such as adhesion junctions, cell adhesion molecules, and steroid synthesis (Supplementary Fig. 12a), further supporting our results. Integrins are important molecules that mediate cell adhesion to the extracellular matrix and can be involved in the regulation of tumor metastatic capacity. Therefore, cytoplasmic and membrane proteins were extracted from BLCA cells transfected with RhoB overexpression plasmids or control plasmids. Western blot analysis was used to detect the integrin β1 and β3 proteins, with GAPDH and ATP1A1 serving as internal references for cytoplasmic and membrane proteins, respectively. The results showed that the integrin β1 protein in the cell membrane was significantly lower in the RhoB-OE group than in the vector group (Fig. 7c and Supplementary Fig. 12b). In addition, the integrin β1 protein in cell membranes was significantly reduced in BLCA cells treated with ZOL in a concentration-dependent manner (Fig. 7d and Supplementary Fig. 12c). Additionally, immunofluorescence staining was used to detect the localization of integrin β1, and the results showed that the abundance of integrin β1 on the cell membrane decreased after RhoB overexpression or MVA pathway inhibition by simvastatin and ZOL in BLCA cells (Fig. 7e, f and Supplementary Fig. 12d, e), consistent with our western blot findings.

We also found that multiple amino acid (e.g., serine, asparagine, etc.) metabolic pathways were altered in RhoB overexpressing T24 cells (Supplementary Fig. 12a, f). Therefore, we initially screened several DEGs related to amino acid metabolism, tumor growth and metastasis from the results of RNA-seq analysis. Asparagine synthetase (ASNS) and 3-phosphoglycerate dehydrogenase (PHGDH) were key enzymes in the asparagine and serine biosynthesis pathways, respectively, and were associated with the growth, progression, and metastasis of various tumors^32,33^. TNFα-stimulated gene-6 (TNFAIP6) could affect the expression of *c-Myc* mRNA^34^, a key transcription factor regulating the proliferation and metastasis of various tumors, including BLCA^35,36^. The changes in mRNA expression of the above genes were verified by qRT-PCR analysis in BLCA cells (T24 and 5637) overexpressing RhoB and the corresponding control cells, and found that the mRNA expression of *ASNS* and *PHGDH* was downregulated, while that of *TNFAIP6* was upregulated after RhoB overexpression (Supplementary Fig. 12g). Meanwhile, analysis of the TCGA-BLCA dataset revealed that *ASNS* mRNA levels were elevated in BLCA tissues compared to normal tissues, while those of *TNFAIP6* mRNA were relatively reduced (Supplementary Fig. 12h). The above evidence suggested that the screened DEGs may play important roles in BLCA progression and metastasis. Our RNA-seq analysis revealed broader transcriptional changes associated with BLCA cell metastasis and growth, and the more critical RhoB-dependent transcription of genes may remain to be further explored and validated.

## Discussion

BLCA metastasis strongly affects patient prognosis. Previous studies have reported that ~50% of muscle-invasive BLCA patients relapse after radical cystectomy, and most relapses involve distant metastases^37^, with common sites of distant metastases, including the lymph nodes, bone, urinary tract, lung and liver^38^. A better understanding of the underlying molecular mechanisms may lead to improved evaluation and treatment of BLCA patients with metastasis.

Dysregulation of lipid metabolism, one of the most prominent metabolic alterations in tumors, significantly contributes to cellular energy storage, metabolism and modulation of multiple signaling molecules^39^. Our group used BLCA tissues and paraneoplastic tissues for transcriptomic assays and found that lipid metabolism is closely related to the development of BLCA^40^. Dysfunction of lipid metabolism in tumor cells is mainly reflected by abnormal activity or expression of metabolic enzymes^41,42^. Previous studies have shown that MVA pathway activation promotes BLCA cell growth and adriamycin resistance^12,43^. Our study revealed that most enzymes within the MVA pathway exhibit highly expressed in BLCA compared to other urologic tumors, with MVA pathway activation correlating with a poorer prognosis for BLCA patients, as evidenced by analyses of single-cell and bulk-transcriptomic datasets.

FDPS is a key enzyme involved in the regulation of the synthesis of intermediate metabolites of the MVA pathway. FPP and GGPP have been reported to participate in the regulation of the biological behavior of various tumors^44–46^. In PTEN-deficient prostate cancer, FDPS plays an important oncogenic role through the GTPase/AKT axis^26^. SREBP-2 and NF-Y can regulate the transcriptional activation of FDPS and thereby regulate the proliferation of hepatoblastoma cells^24^. In addition, p53 has been found to be involved in the transcriptional regulation of several MVA pathway genes, including FDPS^47^. However, few studies have explored the high expression of FDPS in tumors at the level of post-translational modifications. Our study pioneers in revealing the heightened expression of FDPS in BLCA and demonstrates its protein stability regulation via the PSME3-mediated ubiquitin-independent proteasome system.

The intermediate metabolites of the MVA pathway, FPP and GGPP, are required for farnesyl and geranylgeranyl modification, respectively^14^. Moreover, RhoB has been reported to be both geranylgeranylated and farnesylated^30^. In our study, we found that inhibition of the MVA pathway significantly inhibited the migration of BLCA cells and that RhoB protein expression was significantly increased. The addition of GGPP, but not FPP and cholesterol, to the medium significantly reversed the attenuating effect of migration induced by MVA pathway inhibition and resulted in a decrease in RhoB protein expression. In addition, the continued addition of the geranylgeranyltransferase I (GGTase I) inhibitor GGTI298 suppressed the GGPP-induced reversion to migration. The above series of experiments demonstrated that the RhoB protein is a critical effector of the MVA pathway in BLCA cell metastasis and is associated with GGPP-mediated protein geranylgeranylation.

In addition to prenylation, ubiquitination is an important post-translational modification that regulates RhoB protein stability^48^. Previous studies have reported that the E3 ubiquitin ligases Smurf1^49^ and FBXW7^48^, the E3 ligase complex Cullin-3-Rbx1-KCTD10^50,51^, and neddylation-Cullin 2-RBX1^52^ are capable of mediating RhoB degradation via the ubiquitin-proteasome system. In addition, RhoB is degraded via the lysosomal pathway^53^. In our study, we found that RhoB protein degradation was predominantly mediated by the proteasome system in BLCA cells and that MVA pathway inhibition affected the ubiquitination and degradation of the RhoB protein. In addition, RhoB-GG (geranylgeranylated-only RhoB) was more susceptible to ubiquitination than RhoB (both geranylgeranylated and farnesylated RhoB) and RhoB-F (farnesylated-only RhoB). The above results suggest that GGPP-mediated geranylgeranylation may play an important role in the ubiquitination-mediated degradation of the RhoB protein.

Rho GTPases are small GTP/GDP-binding proteins that play important roles in cell migration by affecting actin and myosin activity as well as cell–ECM and cell‒cell adhesion^31^. As an important member of the Rho GTPase family, RhoB is thought to be a negative regulator of cancer progression, invasion and metastasis, as its expression is reduced in a number of tumors, including BLCA^54–58^. Our in vivo and in vitro results also confirmed the ability of RhoB to inhibit the proliferation and metastasis of BLCA cells and that RhoB may act by affecting the translocation of integrin β1 from the cytoplasm to the membrane. During tumor metastasis, tumor cells can interact with the ECM and mediate its remodeling, thus creating a microenvironment conducive to tumor metastasis^59,60^. The integrin β1 family is considered to be a crucial coupling point for cell–ECM interactions, mediating cell adhesion to the ECM and transmitting ECM signals into cells^61,62^. Previous studies reported that downregulation of the cell surface integrin β1 inhibited the metastatic ability of BLCA cells^63^, which further supported our findings.

**Fig. 8:**
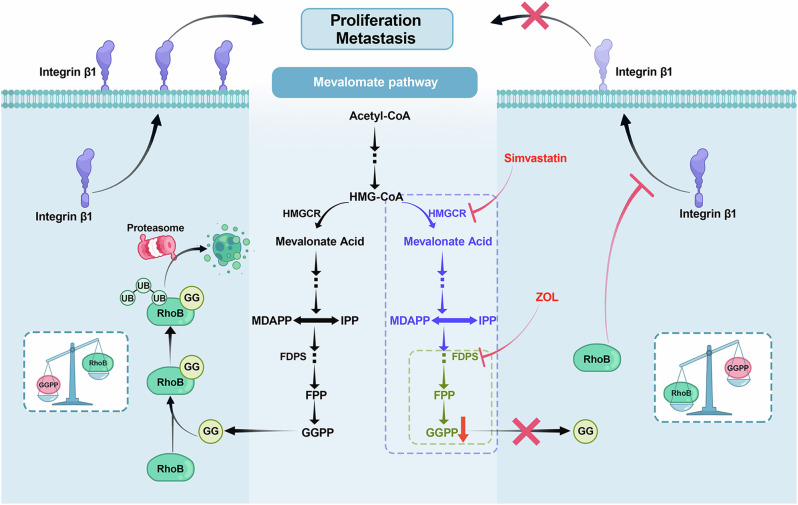
Mechanistic diagram of this study. Activation of the MVA pathway predicts a worse prognosis for patients with BLCA. Inhibition of the MVA pathway by drugs (ZOL or simvastatin) or FDPS knockdown can reduce the content of the intermediate metabolite GGPP, resulting in RhoB proteins that are unable to be geranylgeranylated and degraded. The accumulated RhoB protein inhibits the membrane localization of integrin β1, ultimately suppressing the proliferation and metastasis of BLCA cells. Creation of the illustrations and every element in Fig. 8 was drafted by the authors, and edited by Dr. Yuruo Chen, a diagram editing expert at the Chinese Academy of Science using Adobe Photoshop software. No artificial intelligence or database was involved in the creation of this image.

In conclusion, our study revealed that MVA pathway activation indicates a poorer prognosis in BLCA patients, that FDPS, a key enzyme of the MVA pathway, is highly expressed in BLCA, and that its protein stability is regulated by the PSME3-mediated ubiquitin-independent proteasome system. MVA pathway inhibition by FDPS knockdown or drug treatment (ZOL or simvastatin) significantly inhibited the metastatic ability of BLCA cells both in vitro and in vivo. Further mechanistic studies revealed that RhoB plays an important role in the effect of MVA pathway inhibition on the migratory capacity of BLCA cells and that its protein stability is closely related to GGPP-mediated geranylgeranyl modification (Fig. 8). MREs and their inhibitors may be potential targets and adjuvants for the prevention or treatment of BLCA metastasis.

## Methods

### Human BLCA samples

BLCA tissue samples (*n* = 15) and matched paracancerous tissue samples were obtained from patients with BLCA undergoing radical cystectomy at Zhongnan Hospital of Wuhan University. The clinicopathological features of the BLCA patients are detailed in Supplementary Table 1. The inclusion criteria encompassed BLCA patients requiring radical cystectomy, devoid of metabolism-related diseases, and not taking lipid-lowering drugs or N-BPs. The exclusion criteria included patients with BLCA combined with other tumors, metabolic diseases such as hyperlipidemia and diabetes mellitus, those taking lipid-lowering drugs or N-BPs, and those with secondary bladder tumors. This study was approved by the Ethics Committee of Zhongnan Hospital of Wuhan University (approval number: 2020003). Informed consents were obtained from all subjects to collect the samples. The sample collection and treatment procedures were conducted in accordance with the approved guidelines. All ethical regulations relevant to human research participants were followed.

### Single-cell, bulk-transcriptomic and genetic dataset analysis

Single-cell RNA sequencing (scRNA-seq) data for three BLCA samples (two primary BLCA samples and one recurrent BLCA sample) were obtained from the Gene Expression Omnibus (GEO datasets, https://www.ncbi.nlm.nih.gov/geo/, accession ID: GSE190888). The Seurat package was used for data cleaning and integration. The “FindClusters” function of the Seurat package was used to analyze clustering. Cell types were annotated based on canonical cell type-specific markers^64,65^.

The mRNA expression data, mutation data, copy number alteration data and clinical data of five urologic tumors, including BLCA, kidney chromophobe (KICH), kidney renal clear cell carcinoma (KIRC), kidney renal papillary cell carcinoma (KIRP) and prostate adenocarcinoma (PRAD), which had both tumor and normal samples from the TCGA dataset, were downloaded from the Xena Browser (https://xenabrowser.net/). In this study, edgeR was used to calculate adjusted *P* values and fold changes^66^. Genes with an adjusted *P* value < 0.05 were defined as DEGs. Amplification and deletion heterozygosity were taken into consideration when evaluating the frequency of copy number changes for each gene, with more than five percent considered high-frequency SCNAs. As a measure of the relationship between SCNA and expression, Pearson’s correlation was calculated between expression values and copy number segment values for each gene.

### Establishing the MVA potential index (MPI) model

The index represents the level of MVA pathway activity based on the expression data for genes encoding enzymes related to the MVA pathway such as *ACAT1, ACAT2, FDFT1, FDPS, GGPS1, HMGCL, HMGCR, HMGCS1, IDI1, IDI2, MVD, MVK* and *PMVK*. We calculated the enrichment score (ES) for each gene set by utilizing the single sample gene set enrichment analysis (ssGSEA) package provided by the R package “GSVA”^67^, defined as the MVA potential index (MPI). Since the MVA pathway inhibition by atorvastatin treatment or *FDPS* knockdown was unequivocal, we selected two independent datasets (GSE2450 and GSE252007) to validate the ability of MPI to represent the level of MVA pathway activity in cells. The MPI in BLCA tissues and normal tissues was also validated using independent BLCA gene expression datasets (GSE40355, GSE3167 and TCGA-BLCA dataset). Finally, the samples (TCGA-BLCA dataset and GSE13507) were divided into two groups (high MPI and low MPI) based on the MPI, consisting of 30% of the top 30% and 30% of the bottom 30%, for overall survival analysis of patients with BLCA.

### Cell lines and chemicals

The human BLCA cell lines T24 (Cat. #TCHu 55), 5637 (Cat. #TCHu 1), and UM-UC-3 (Cat. #TCHu217) and HEK 293 T (Cat. #GNHu17) were obtained from the Chinese Academy of Sciences (Shanghai, China) and were identified by Cell Bank, Chinese Academy of Sciences (Shanghai, China). All cell lines were verified using short tandem repeat (STR) assays and no mycoplasma was detected. The T24 and 5637 cell lines were cultured in RPMI-1640 medium supplemented with 10% fetal bovine serum (FBS). The UM-UC-3 and HEK 293T cell lines were cultured in DMEM (containing 10% FBS).

ZOL (Cat. #HY-13777), simvastatin (Cat. #HY-17502) and cholesterol (Cat. #HY-N0322A) were purchased from MCE. FPP (Cat. #116057-57-9) and GGPP (Cat. #313263-08-0) standards were purchased from Cayman Chemical.

### siRNAs and plasmids

The siRNAs targeting *FDPS* and *RhoB* used in this study were purchased from GenePharma (Suzhou, China). The siRNA sequences are shown in Supplementary Table 2.

The Flag-RhoB-GG and Flag-RhoB-F plasmids were constructed according to Baron et al.^30^. The human shFDPS lentiviral vector, human RhoB overexpression lentiviral vector and negative control vector were purchased from GenePharma (Suzhou, China). The other plasmids were constructed through standard subcloning techniques. DNA sequencing was performed to confirm the RNA integrity. The siRNAs and plasmids were transfected into cells with Opti-MEM culture medium, Lipofectamine® 3000 and P3000™ (Invitrogen).

### MTT assay

Two hundred microliters of medium were suspended in 3000 BLCA cells, which were seeded in 96-well plates for the indicated times. After 20 μL of 5 mg/mL MTT was added to each well and incubated for 4 h at 37 °C, 150 μL of DMSO was added to each well. The absorbance of each well at 570 nm was measured using a microplate reader (Molecular Devices, USA) to assess cell viability.

### Clonogenic survival assay

After transfection or drug treatment, 2 mL of medium was suspended in 1000 BLCA cells, which were seeded in 6-well plates and cultured until the colonies emerged and grew to the appropriate size. The medium was removed, and the cells were fixed and stained with 4% paraformaldehyde (PFA) and 0.1% crystal violet solution. Cell viability was judged according to the number of clones in each well.

### Transwell chamber migration assay

After transfection or drug treatment, a total of 8 × 10^4^ 5637 cells, 4 × 10^4^ T24 cells, and 4 × 10^4^ UM-UC-3 cells were suspended in 200 μL of serum-free medium. The suspension was seeded in the upper transwell chamber, and 600 μL of medium containing 10% FBS was added to the lower chamber. After incubating at 37 °C for 24 h, the cells were fixed and stained with 4% PFA and 0.1% crystal violet solution. Then, a phase contrast microscope was used to photograph and count the cells that had migrated.

### Wound healing assay

BLCA cells were seeded in 6-well plates. When the cell confluence reached 100%, we scratched the cells with a 200 μL pipette tip. Then, the cells were washed twice with PBS, followed by the addition of medium containing different concentrations (0, 10 and 20 μM) of ZOL. The cells were photographed by phase contrast microscopy at 0 and 24 h for several pre-marked spots.

### Measurement of FPP and GGPP

Drug-treated BLCA cells and control cell samples were collected, and 300 μL of extract (containing acetonitrile, methanol and 0.1 M formic acid) was added. The samples were incubated at −20 °C for 10 min after 30 s of vortex shaking and then centrifuged at 12,000 r/min for 5 min at 4 °C. The supernatant was subsequently collected and subjected to LC–MS/MS analysis. The extraction, metabolite identification and quantification were performed at Wuhan MetWare Biotechnology Co., Ltd. (Wuhan, China) following their standard procedures. All the chemicals were analytical reagent grade. Authentic FPP and GGPP standards were purchased from Cayman Chemical.

### Measurement of total cholesterol

Total cholesterol was assayed using the Amplex Red Cholesterol Test Kit (Cat. #S0211S, Beyotime) following the manufacturer’s protocol. Briefly, drug-treated BLCA cells and control cell samples (5 × 10^5^ cells) were collected. Cells were lysed by the addition of 100 μL of BeyoLysis™ Buffer A. The cells were subsequently centrifuged at 12,000 × *g* for 3–5 min at 4 °C, after which the supernatant was removed for subsequent assays. Cholesterol assay buffer, Amplex Red, cholesterol esterase and enzyme mixture were added to the supernatant, and the mixture was allowed to react for 30 min at 37 °C under light protection. The absorbance of each well at 570 nm was measured using a microplate reader (Molecular Devices, USA).

### Total RNA isolation

Total RNA was isolated from BLCA cells with a HiPure Total RNA Mini Kit from Magen (Cat. #R4111-03) according to the manufacturer’s protocol. The quantity of isolated RNA was assessed with a NanoDrop^®^ ND-2000 UV–Vis spectrophotometer (Thermo Scientific, USA).

### Quantitative reverse transcription PCR (qRT-PCR)

Reverse transcription was conducted to synthesize cDNA according to the instructions of the ReverTra Ace qPCR RT Kit (Cat. #FSQ-101, Toyobo). cDNA (500 ng) was used for each PCR in a final volume of 15 μL. The values were normalized to the GAPDH amplification values. The primer sequences are shown in Supplementary Table 3.

### RNA sequencing

T24 cells were transfected with siNC or siFDPS-1, or with RhoB overexpression plasmids and control plasmids. Subsequently, the cells were collected for RNA extraction. The extracted RNA samples were subsequently sent to Bioprofile (Shanghai, China) for sequencing. The DEGs identified via RNA-seq were analyzed with the R package “DEseq2”, and the screening criterion was adjusted to *p* < 0.05. The RNA sequencing data have been deposited into the NCBI GEO under the accession numbers GSE252007 and GSE270394.

### Proteomics

T24 cells treated with ZOL (20 μM) or simvastatin (5 μM) for 40 h were collected, and control cells were used. The reaction mixture (1% SDC/100 mM Tris-HCl, pH = 8.5/10 mM TCEP/40 mM CAA) was added to the sample and incubated at 60 °C for 1 h to complete protein denaturation, reduction and alkylation. Trypsin was added at a ratio of 1:50 (enzyme:protein, w/w) overnight digestion at 37 °C. TFA was used to bring the pH down to 6.0 to end the digestion. After centrifugation (16,000 × *g*, 15 min), the supernatant was subjected to peptide purification. All the samples were analyzed on a timsTOF Pro (Bruker Daltonics) hybrid trapped ion mobility spectrometer (TIMS) quadrupole time-of-flight mass spectrometer. An UltiMate 3000 RSLCnano system (Thermo Fisher Scientific, USA) was coupled to a timsTOF Pro with a CaptiveSpray nanoion source (Bruker Daltonics, USA). DIA raw data were analyzed with DIA-NN (V1.8.1). The spectral files were searched against the human protein sequence database downloaded from UniProt. A library-free search was performed according to the DIA-NN manual (https://github.com/vdemichev/DiaNN/). A predicted in silico spectral library was generated from the FASTA database. The false discovery rate (FDR) was set to 0.01 for reliable precursor identification. The “MBR” was enabled. Protein intensities were normalized with the MaxLFQ algorithm. The mass spectrometry proteomics data have been deposited in the ProteomeXchange Consortium via the iProX partner repository^68,69^ with the dataset identifier PXD048067 (https://www.iprox.cn/page/project.html?id=IPX0007786000).

### Immunoprecipitation–mass spectrometry (IP–MS) analysis

293T cells were transfected with the Flag-Vector or Flag-FDPS plasmid for 48 h, followed by immunoprecipitation. Magnetic beads were collected, and the reaction solution was added for reduction, alkylation and elution. Trypsin was added for enzymatic hydrolysis overnight. After enzymatic hydrolysis, the peptide solution was desalted by passing through a desalting column. The peptide samples were then centrifuged and purified. Mass spectrometry analysis was performed using an Orbitrap Exploris 480 Liquid Chromatography–Mass Spectrometry (LC–MS) system (Thermo Fisher Scientific, USA). The results generated by LC–MS were retrieved by MaxQuant (v1.6.2.10) with the database retrieval algorithm MaxLFQ.

### Isolation of total protein, membrane and cytoplasmic protein

The cells were collected and lysed with RIPA buffer (containing protease inhibitor and phosphatase inhibitor) on ice for 30 min. The cell lysates were centrifuged at 12,000 × *g* for 10 min to collect the supernatant. The supernatant was diluted in 5× sampling buffer, heated at 100 °C for 10 min, and stored at −80 °C for subsequent experiments. A Minute™ Plasma Membrane Protein Isolation Kit (Cat. #SM-005, INVENT) was used to isolate the membrane and cytoplasmic proteins from the BLCA cells according to the manufacturer’s protocol.

### Western blots

The proteins were separated using 7.5–12.5% SDS–PAGE gels and then transferred to PVDF membranes (Millipore, USA). The membranes were blocked in 5% TBST fat-free milk for 2 h at room temperature. The membranes were cut horizontally according to the protein marker instructions (Shanghai Epizyme, China) and incubated separately with the appropriate primary antibodies (Supplementary Table 2) overnight at 4 °C. After washing three times with TBST, the membranes were incubated with secondary antibody for 2 h at room temperature. The bands were detected using an enhanced chemiluminescence kit (Bio-Rad, USA), and the blots were exposed to a BioSpectrum Gel Doc-IT2 315 167 Imaging System (UVP, USA). The specific primary antibodies used are shown in Supplementary Table 4.

### Co-immunoprecipitation (Co-IP) assays

The Co-IP assay was performed using the BeaverBeads Protein A Immunoprecipitation Kit (Cat. #22202-20, BEAVER) according to the manufacturer’s protocol. Briefly, 20 µL of magnetic beads was incubated with 1 µg of the target antibody for more than 4 h at 4 °C. Then, the cell lysates were added to the antibody-magnetic bead complex and incubated overnight at 4 °C. After the immunoprecipitation reaction, the magnetic beads coupled with the complexes were washed three times with Triton X-100 buffer. The protein-antibody-bead complexes were resuspended in 50 μL of 1× SDS loading buffer and denatured in a water bath at 100 °C for 10 min for further immunoblot analysis. The specific primary antibodies used are shown in Supplementary Table 4.

### Immunofluorescence staining

After transfection or drug treatment, the BLCA cells were plated on 12-mm coverslips and incubated for 12 h. After the cells had adhered, the coverslips were washed twice with PBS, fixed with 4% PFA for 30 min, and washed with PBS again. The cells were then subjected to a series of steps, including blocking and antibody incubation. Finally, the cells were stained for immunofluorescence and observed. Images were captured using a laser confocal microscope (Nikon C2^+^, Japan). The details of the antibodies used in this study are listed in Supplementary Table 4.

### Xenograft model and pulmonary metastasis model

The animal experiment was approved by and performed under the regulations of the Experimental Animal Welfare and Ethics Committee at Zhongnan Hospital of Wuhan University (approval number: ZN2023114). We have complied with all relevant ethical regulations for animal use.

Four-week-old male BALB/c-nude mice were purchased from Beijing Vital River Laboratory Animal Technology Co., Ltd. (Beijing, China). All mice were housed under specific pathogen-free conditions in a controlled environment (temperature, 20–24 °C; relative humidity, 30–70%; and 12-h light–dark cycle) and allowed unrestricted access to food and water at the Animal Experiment Center of Zhongnan Hospital of Wuhan University. The xenograft model was established by subcutaneous injection of 150 μL of PBS solution containing 2 × 10^7^ cells in the dorsal region near the forelimb of mice. Six days later, the mice were randomly divided into two groups. The experimental group (*n* = 3) was treated with ZOL, which was dissolved in PBS, intraperitoneally at a dose of 100 μg/kg, while the control group (*n* = 3) was intraperitoneally injected with equal amounts of PBS. The drug was administered three times a week for 4 weeks. The tumor size was measured with a Vernier caliper and calculated according to the formula (tumor size = length × width^2^ × 0.5 mm^3^) at regular intervals. According to the regulations of the Experimental Animal Welfare and Ethics Committee at Zhongnan Hospital of Wuhan University, the maximum length of tumors should not exceed 20 mm or the volume of tumors should not exceed 2000 mm^3^; therefore, all experiments in this study did not exceed these limits. The mice were sacrificed by cervical dislocation, and the tumors were collected. After the weights were measured, the tumor tissues were fixed in 4% PFA in preparation for subsequent staining.

For the pulmonary metastasis model, 100 μL of PBS solution containing 1 × 10^6^ cells was injected into the tail vein of mice. Six weeks later, the lungs of the nude mice were observed using a small animal in vivo imaging system Xtreme BI (Bruker, Germany), the mice were subsequently sacrificed by cervical dislocation, and the lung tissues were removed and fixed with 4% PFA for subsequent staining. The pulmonary metastasis model treated with ZOL was used as a xenograft model.

### Immunohistochemical staining for bladder tissue samples

Tissue microarray (containing 68 BLCA specimens and 40 paracancerous tissues) was collaborated with Shanghai Outdo Biotech (Shanghai, China). The paraffin sections were dewaxed, subjected to antigen retrieval, blocking, and incubation. Finally, DAB solution was added, and the sections were placed under a microscope for observation and imaging. The details of the antibodies used in this study are listed in Supplementary Table 4. The expression of FDPS in the bladder tissues from the tissue microarray was blindly quantified by pathologist. The staining scores of FDPS expression is the intensity of staining (0 = negative, 1 = weak, 2 = moderate, and 3 = strong) multiplied by the percentage of positive cells (0 = negative), (1 = 1%–25%), (2 = 26%–50%), (3 = 51%–75%), (4 = 76%–100%), which was performed as our previous described^70^. The patients were divided into a high FDPS protein level group and a low FDPS protein level group according to the median staining scores of FDPS expression.

### Hematoxylin and eosin (H&E) staining

The samples were sequentially processed with xylene, graded alcohol (100%, 96%, 80%, 70% ethanol) and H_2_O. Then, 10% hematoxylin was added for 7 min, after which the samples were washed with water. Then, 1% eosin and 0.2% glacial acetic acid were applied to the cytoplasm for only seconds, after which the cells were washed with water again. Then, the samples were dehydrated in graded alcohol (70%, 80%, 96%, 100% ethanol) and xylene. Finally, an inverted phase contrast microscope (Leica, Germany) was used to obtain images.

### Statistics and reproducibility

All the statistical analyses were performed with R and GraphPad Prism software. The data were assumed to be normally distributed, but this assumption was not formally tested. The statistical tests of the two groups of data were analyzed using a two-tailed paired/unpaired Student’s *t-*test. For data from more than two groups, one-way ANOVA with Tukey’s correction was used. Survival analysis was performed with the log-rank test. The data are presented as the mean ± standard deviation (S.D.). For all the statistical tests, *p* value < 0.05 was considered to indicate statistical significance.

The sample size (*n*) and statistical significance are shown in the figures. No specific statistical methods were used to predetermine the sample size. Each key in vitro finding was replicated with at least two cell lines. For animal studies, we used at least three replicates. RNA-seq and proteomics samples were analyzed by independent investigators blinded to the experimental conditions/treatments. The expression of FDPS in the bladder tissues from the tissue microarray was blindly quantified by pathologist. For all the other studies, the data collection and analysis were not performed while the participants were blinded to the conditions of the experiments.

### Reporting summary

Further information on research design is available in the Nature Portfolio Reporting Summary linked to this article.

## Supplementary information

Supplementary Information

Description of Additional Supplementary Files

Supplementary Data 1

Supplementary Data 2

Reporting Summary

## Data Availability

The mass spectrometry proteomics data have been deposited in the ProteomeXchange Consortium via the iProX partner repository with the dataset identifier PXD048067 (https://www.iprox.cn/page/project.html?id=IPX0007786000). The RNA sequencing data have been deposited into the GEO database with the accession numbers GSE252007 and GSE270394. The results of the IP–MS assays generated in this study are provided in Supplementary Data 1. The publicly available source data are accessible from their respective publications^23,71–75^ and can be found in the GEO database under accession numbers GSE13507, GSE32548, GSE190888, GSE3167, GSE40355 and GSE2450. TCGA-BLCA data were obtained from the Xena Browser (https://xenabrowser.net/). The remaining data can be accessed in the article or in the Supplementary Information. The Supplementary Information file contains all Supplementary Figs. (Supplementary Figs. 1–12) and the original uncropped western blots (Supplementary Fig. 13). Source data are provided in Supplementary Data 2.
