# Supplementary material for: Mevalonate pathway inhibition reduces bladder cancer metastasis by modulating RhoB protein stability and integrin β1 localization
Source: Commun Biol. 2024 Nov 9;7:1476. doi: 10.1038/s42003-024-07067-8 (PMC11550803; doi:10.1038/s42003-024-07067-8)
Supplement: Supplementary file 1 — Supplementary Information [file 42003_2024_7067_MOESM1_ESM.pdf]

Supplementary Information

Mevalonate pathway inhibition reduces bladder cancer metastasis by modulating RhoB protein stability and integrin β1 localization

Supplementary Figures 1-13: Pages 2-34

Supplementary Tables 1-4: Pages 35-38

Supplementary Figures 1-13

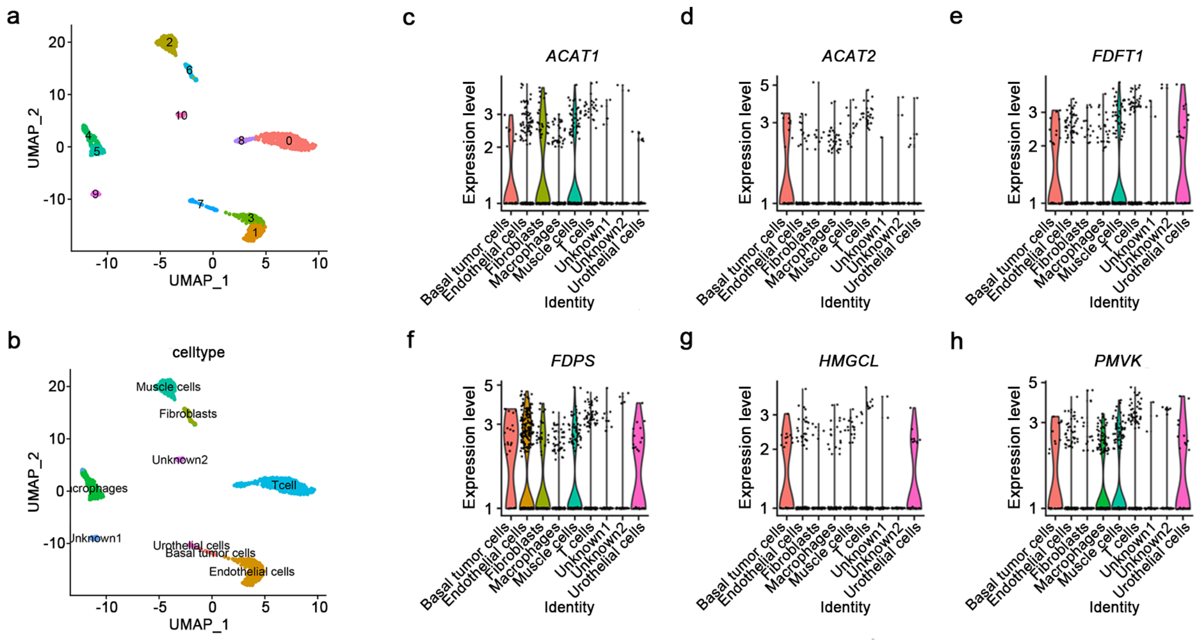

Supplementary Figure 1. MREs are upregulated in basal tumor cells and urothelial cells. **a.** UMAP-projection of scRNA sequencing data for three BLCA samples were obtained from the GEO datasets (*https://www.ncbi.nlm.nih.gov/geo/*, accession ID: GSE190888). **b.** Cell types were annotated based on canonical cell type-specific markers. basal tumor cells, endothelial cells, fibroblasts, macrophages, muscle cells, T cells, and urothelial cells. **c-h.** MREs (*ACAT1*, *ACAT2*, *FDFT1*, *FDPS*, *HMGCL*, and *PMVK*) expression in each major cell population. *ACAT1* (**c**), *ACAT2* (**d**), *FDFT1* (**e**), *FDPS* (**f**), *HMGCL* (**g**), and *PMVK* (**h**).

Supplementary Figure 2

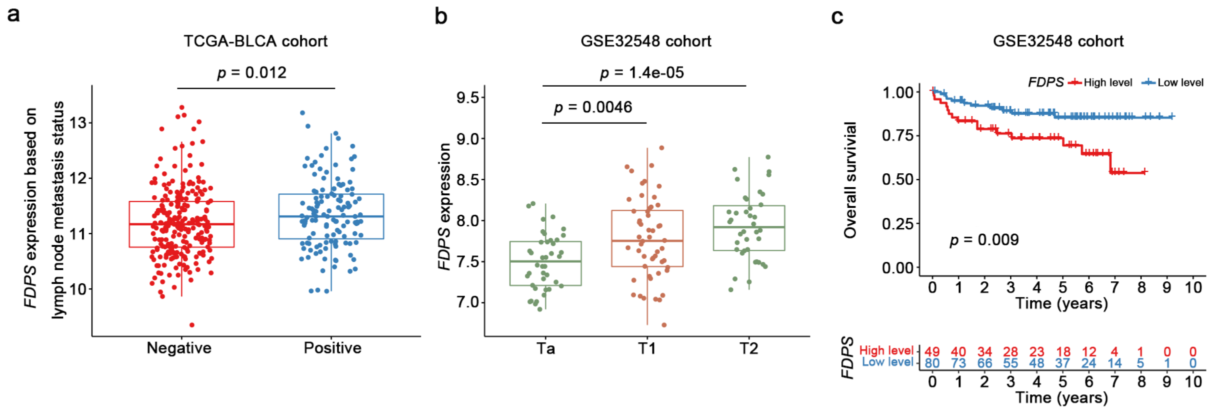

Supplementary Figure 2. FDPS is upregulated in BLCA.

a. The mRNA level of *FDPS* in patients with different lymph node metastasis statuses (negative and positive) in the TCGA-BLCA cohort (RNA-seq data). **b.** The mRNA expression level of *FDPS* in BLCA with different T stages (Ta, T1, and T2) in the GSE32548 cohort (RNA-seq data). **c.** OS analysis of patients with BLCA who had different *FDPS* mRNA levels in the GSE32548 dataset. Statistical significance was ascertained by two-tailed unpaired Student's t-tests (a and b) and the log-rank test of Kaplan-Meier analysis (c).

Supplementary Figure 3

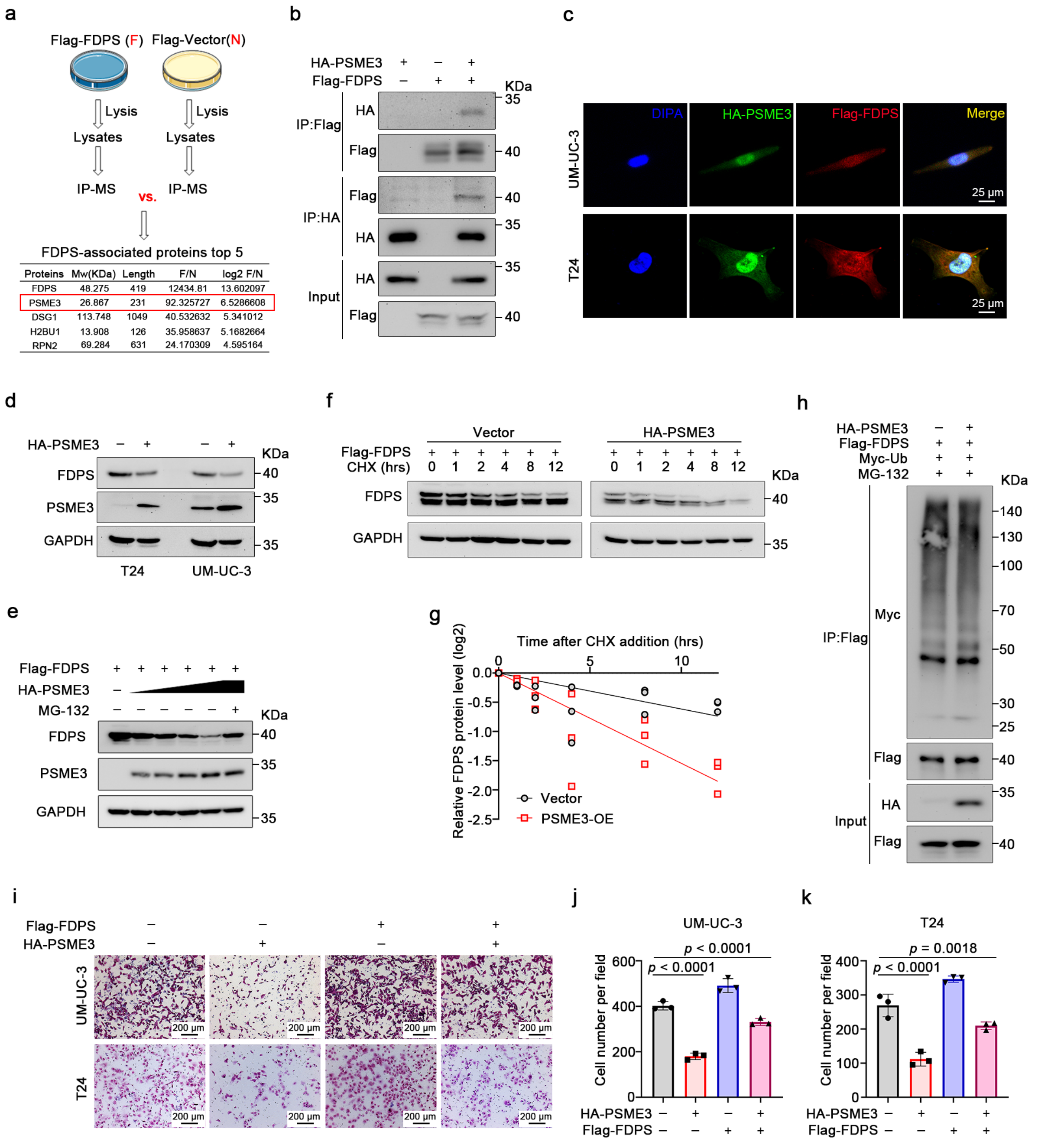

Supplementary Figure 3. PSME3 regulates the stability of FDPS proteins.

a. Flow chart analyzing the proteins interacting with FDPS in 293T cells by IP-MS. **b.** 293T cells were transfected with the HA-PSME3 and Flag-FDPS plasmids for 48 hrs, and Co-IP was performed with anti-Flag or anti-HA antibodies. **c.** HA-PSME3 and Flag-FDPS plasmids were transfected into T24 and UM-UC-3 cells for immunofluorescence assays. The scale bar is 25 μm. **d.** Western blotting was performed to detect FDPS protein levels after PSME3 was

overexpressed in T24 and UM-UC-3 cells. **e.** 293T cells were transfected with the described plasmids for 48 hrs and then treated with or without MG132 (10 μM) for 8 hrs, followed by Western blotting. **f-g.** 293T cells were transfected with the described plasmids for 48 hrs, treated with CHX (50 μg/ml), and then harvested at the indicated time points. The statistical plot (**g**) represents the intensity of the FDPS bands detected by Western blot (n = 3). **h.** 293T cells were transfected with the described plasmids for 48 hrs and then treated with MG132 (10 μM) for 8 hrs. Ubiquitination assays were performed to study the effect of PSME3 on FDPS ubiquitination. **i-k.** BLCA cells (UM-UC-3 and T24) were transfected with the described plasmids for 48 hrs. Transwell migration assays were performed to detect changes in cell migration ability (**i**) (n = 3). The scale bar is 200 μm. (**j** and **k**) Statistical analysis of the transwell migration assay results. The n number represents n biologically independent experiments in each group. Statistical significance was ascertained by one-way ANOVA with Dunnett's multiple comparisons test (j and k). The data are shown as the mean ± SD.

Supplementary Figure 4

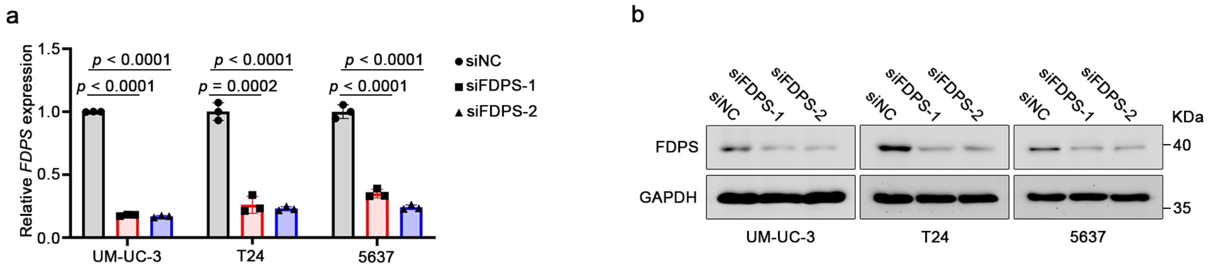

Supplementary Figure 4. Validation of the knockdown efficiency of FDPS siRNAs.

a-b. Validation of the knockdown efficiency of two FDPS-specific siRNAs by qRT-PCR (n = 3) (**a**) and Western blot (**b**). Statistical significance was ascertained by one-way ANOVA with Dunnett's multiple comparisons test (a). The data are shown as the mean ± SD.

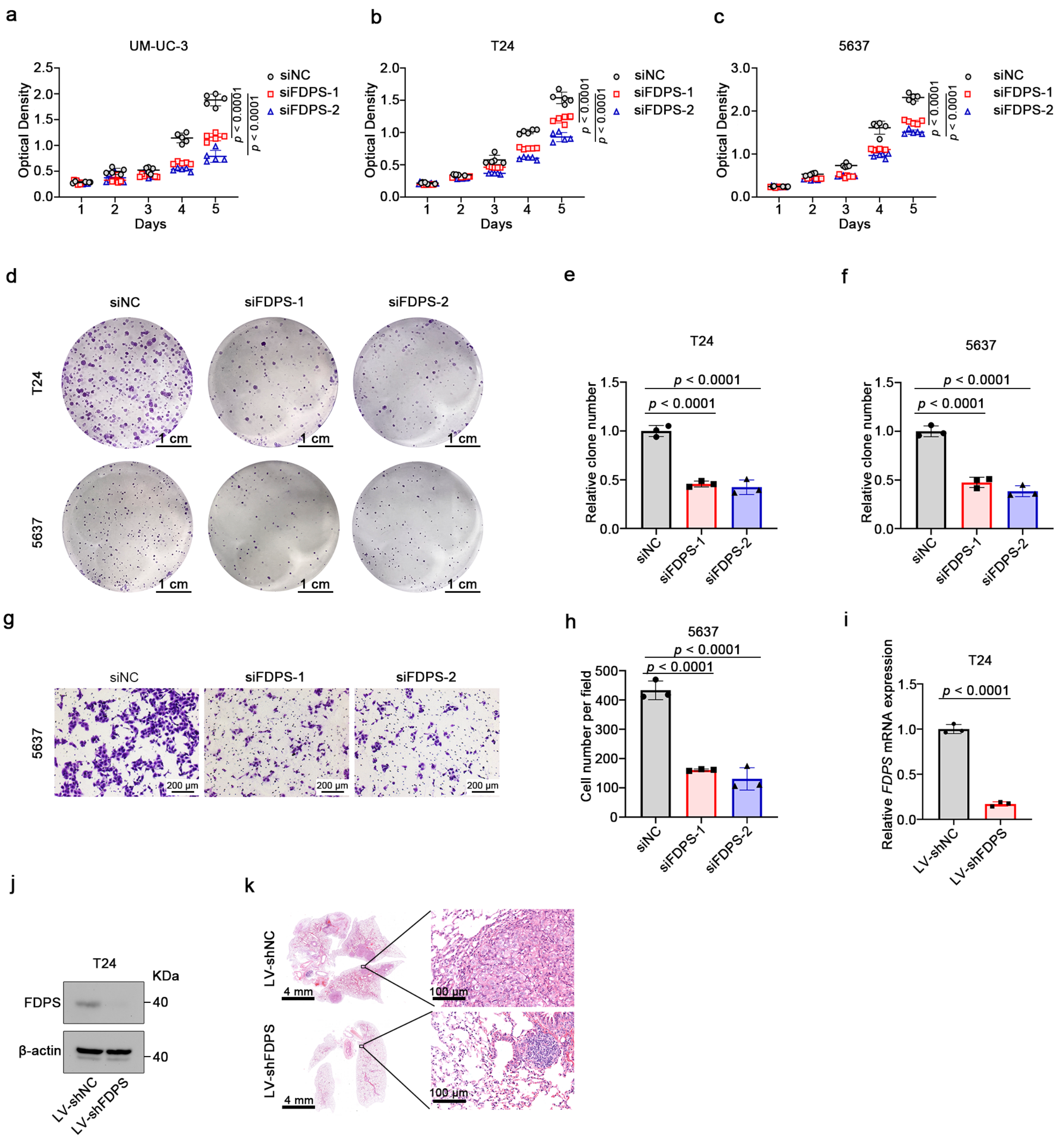

Supplementary Figure 5. FDPS promotes BLCA proliferation and metastasis.

a-c. The cell proliferation curves of BLCA UM-UC-3 (**a**), T24 (**b**) and 5637 (**c**) cells with *FDPS* knockdown determined by MTT assay (n = 5). **d-f.** Representative images (**d**) and statistical analysis (**e-f**) of colony formation assays in T24 and 5637 cells with *FDPS* knockdown (n = 3). The scale bar is 1 cm**. g-h.** Representative images (**g**) and statistical graph (**h**) of transwell migration assays from the indicated groups of 5637 cells with *FDPS* knockdown (n = 3). The scale bar is 200 μm**. i-j.** Validation of the FDPS overexpression efficiency of the FDPS plasmid by qRT-PCR (**i**) and Western blot (**j**). **i-j.** The knockdown

efficiency of *FDPS* in T24 LV-shFDPS cells was verified by qRT-PCR (**i**) and Western blot (**j**). **k**. Images of dissected whole lungs after T24-shNC or T24-shFDPS cells were injected into the tail veins of BALB/C-nude mice for six weeks. The scale bars are 4 mm and 100 μm. The *n* number represents n biologically independent experiments in each group. Statistical significance was ascertained by two-tailed unpaired Student's t-test (i) and one-way ANOVA with Dunnett's multiple comparisons test (a, b, c, e, f, and h). The data are shown as the mean ± SD.

Supplementary Figure 6

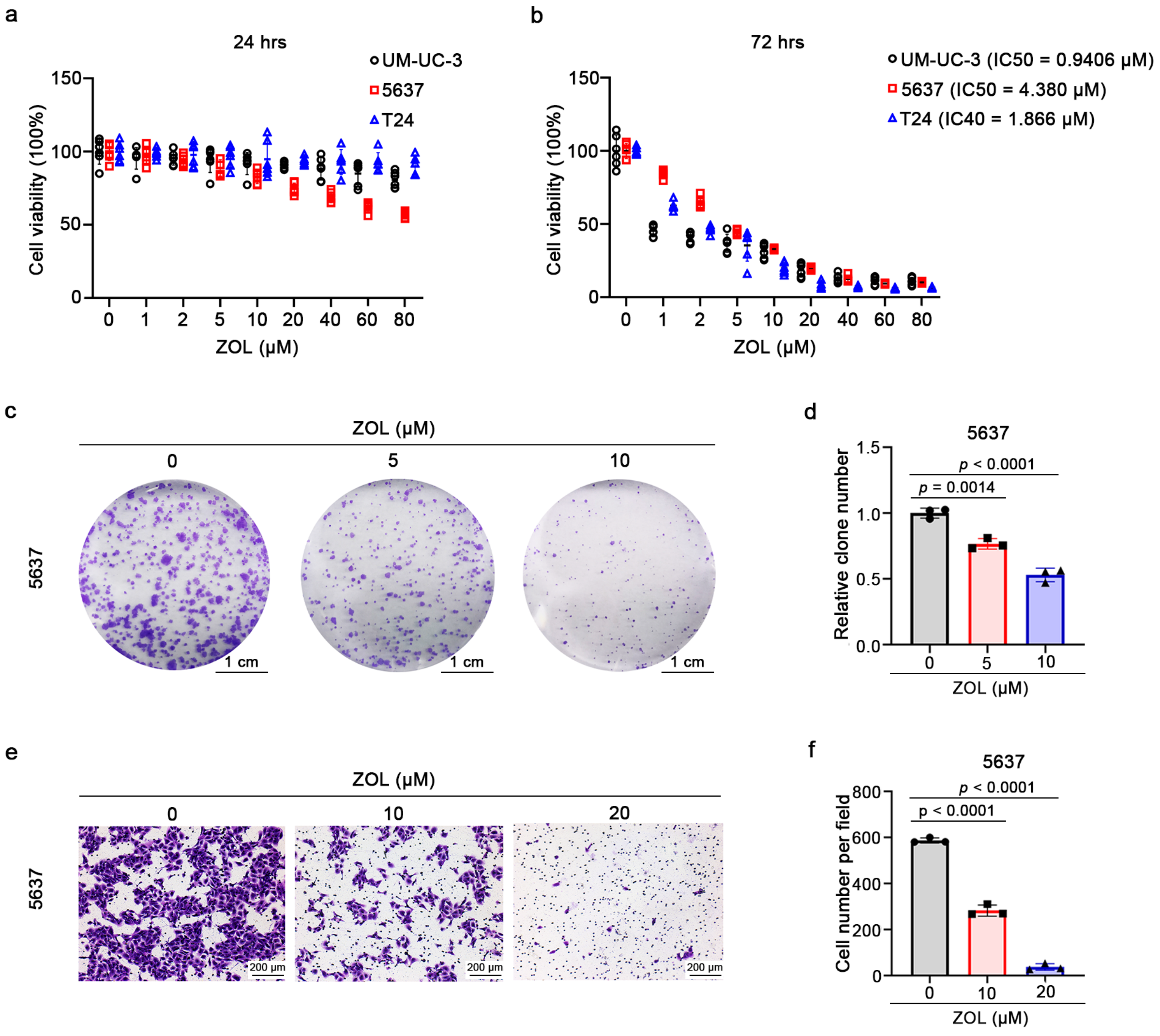

Supplementary Figure 6. ZOL-mediated inhibition of the mevalonate pathway affects BLCA proliferation and metastasis.

a-b. MTT assay was performed to detect the changes in the proliferation ability of BLCA cells (UM-UC-3, T24 and 5637) after treatment with different concentrations (0, 1, 2, 5, 10, 20, 40, 60, and 80 μM) of ZOL for 24 hrs (**a**) or 72 hrs (**b**) (n = 6). **c-d.** Representative images (**c**) and statistical analysis (**d**) of colony formation assays from the indicated groups of 5637 cells after treatment with ZOL at different concentrations (0, 10, and 20 μM) for 48 hrs (n = 3). The scale bar is 1 cm. **e-f.** Representative images (**e**) and statistical graph (**f**) of transwell assays of 5637 cells from the indicated groups after treatment with ZOL at different concentrations (0, 10, and 20 μM) for 48 hrs (n = 3). The scale bar is 200 μm. The n number represents n biologically

independent experiments in each group. Statistical significance was ascertained by one-way ANOVA with Dunnett's multiple comparisons test (d and f). The data are shown as the mean ± SD.

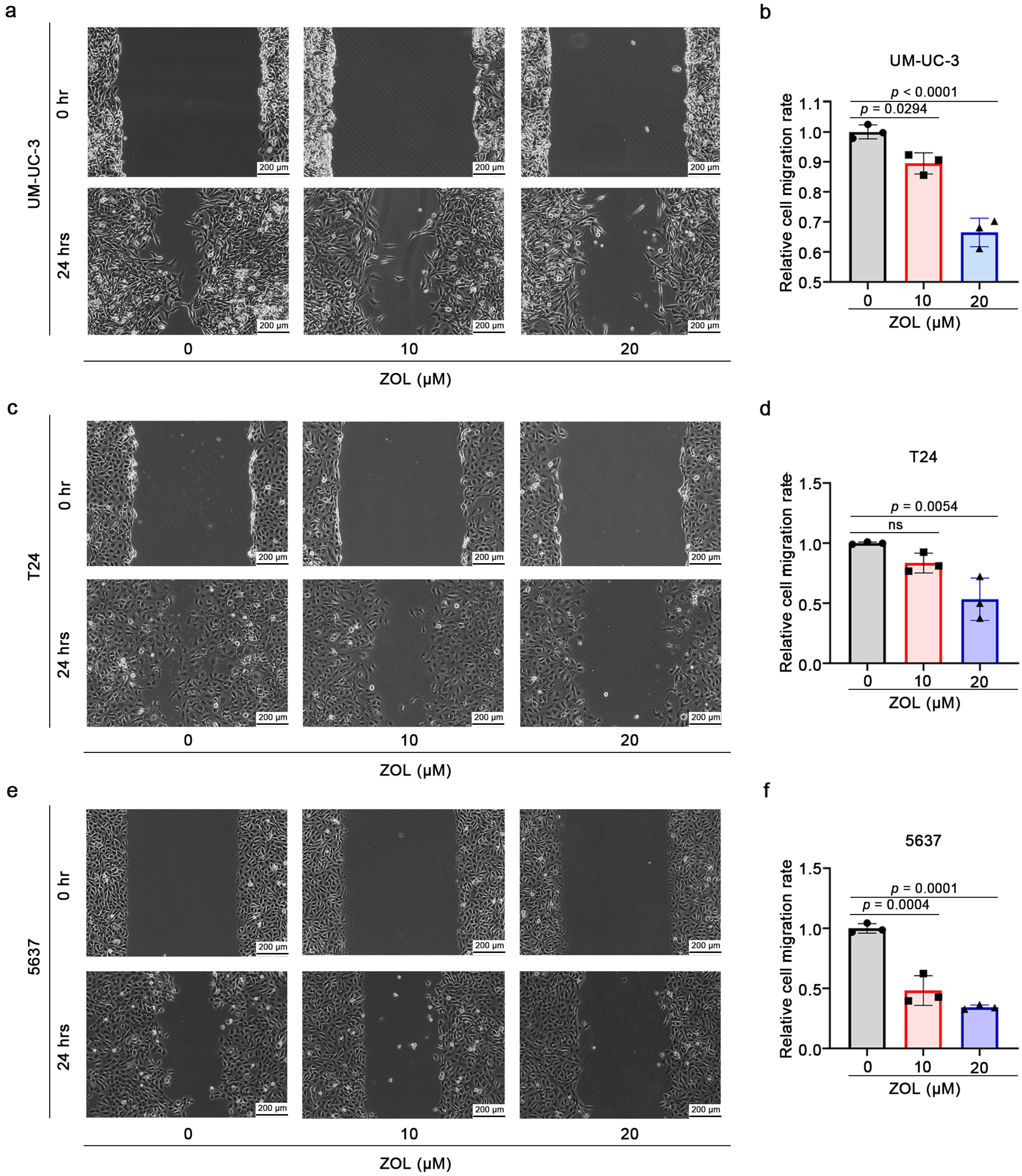

Supplementary Figure 7. ZOL-mediated inhibition of the mevalonate pathway affects BLCA metastasis.

a-f. BLCA cells were treated with different concentrations (0, 10, and 20 μM) of ZOL for 48 hrs. Wound healing assays were performed to detect changes in the migration ability of BLCA UM-UC-3 (a), T24 (c) and 5637 (e) cells ($n = 3$). The statistical graph (b, d and f) of the wound

healing assays. The *n* number represents n biologically independent experiments in each group (n = 3). The scale bar is 200 µm. Statistical significance was ascertained by one-way ANOVA with Dunnett's multiple comparisons test (b, d and f). The data are shown as the mean ± SD.

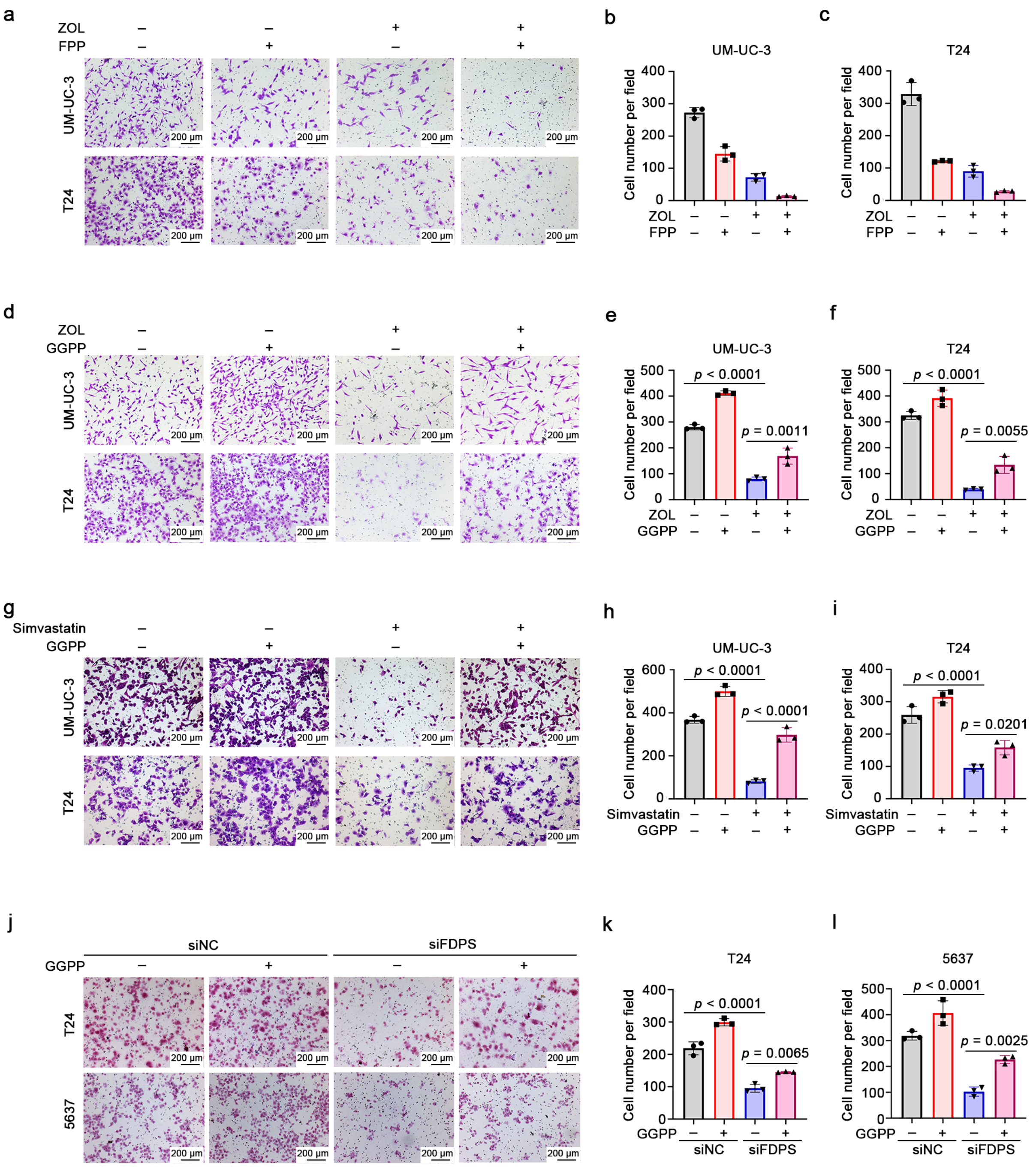

Supplementary Figure 8. GGPP can reverse the inhibitory effect of mevalonate pathway inhibition on migration.

a-c. Representative images (**a**) and statistical graph (**b** and **c**) of transwell migration assays from the indicated groups after treatment of BLCA cells (UM-UC-3 and T24) with ZOL (20 μM) and FPP (5 μM), respectively, or in combination (n = 3). The scale bar is 200 μm. **d-f.** Representative images (**d**) and statistical analysis (**e** and **f**) of transwell migration assays from

the indicated groups after treatment of BLCA cells (UM-UC-3 and T24) with ZOL (20 μM) and GGPP (5 μM), respectively, or in combination (*n* = 3). The scale bar is 200 μm. **g-i**. Representative images (**g**) and statistical analysis (**h** and **i**) of transwell migration assays from the indicated groups after treatment of BLCA cells (UM-UC-3 and T24) with simvastatin (5 μM) and GGPP (5 μM), respectively, or in combination (*n* = 3). The scale bar is 200 μm. **j-l**. Representative images (**j**) and statistical analysis (**k** and **l**) of transwell migration assays from the indicated groups after treatment of BLCA cells (UM-UC-3 and T24) with FDPS siRNA and GGPP (5 μM), respectively, or in combination (*n* = 3). The scale bar is 200 μm. The *n* number represents n biologically independent experiments in each group. Statistical significance was ascertained by one-way ANOVA with Dunnett's multiple comparisons test (e, f, h, i, k, and l). The data are shown as the mean ± SD.

Supplementary Figure 9

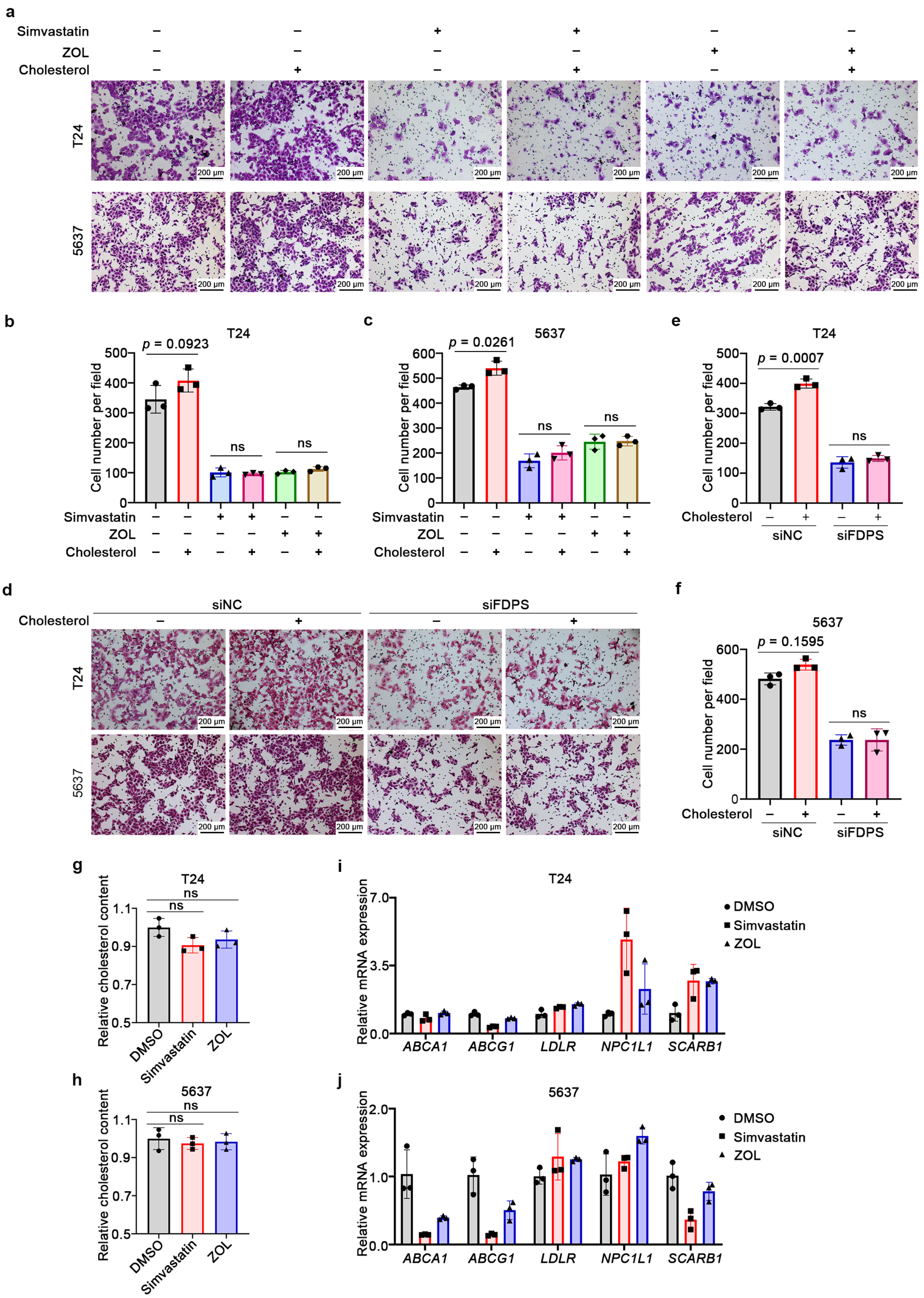

Supplementary Figure 9. Cholesterol cannot reverse the inhibitory effect of mevalonate pathway inhibition on migration.

Supplementary Figure 9

a-c. Representative images (**a**) and statistical graph (**b** and **c**) of transwell migration assays from the indicated groups after treatment of BLCA cells (T24 and 5637) with ZOL (20 μM), simvastatin (5 μM) and cholesterol (25 μM), respectively, or in combination (*n* = 3). The scale bar is 200 μm. **d-f**. Representative images (**d**) and statistical analysis (**e** and **f**) of transwell migration assays from the indicated groups after treatment of BLCA cells (T24 and 5637) with FDPS siRNA and cholesterol (25 μM), respectively, or in combination (*n* = 3). The scale bar is 200 μm. **g-h.** The cholesterol contents in T24 (**g**) and 5637 (**h**) cells treated with ZOL or simvastatin (*n* = 3). **i-j.** The mRNA expression levels of genes related to cholesterol uptake (*LDLR*, *NPC1L1*, and *SCARB1*) and efflux (*ABCA1* and *ABCG1*) in simvastatin- or ZOL-treated BLCA T24 (**i**) and 5637 (**j**) cells were measured by qRT-PCR (*n* = 3). The *n* number represents n biologically independent experiments in each group. Statistical significance was ascertained by one-way ANOVA with Dunnett's multiple comparisons test (b, c, e, f, g, and h). The data are shown as the mean ± SD.

Supplementary Figure 10

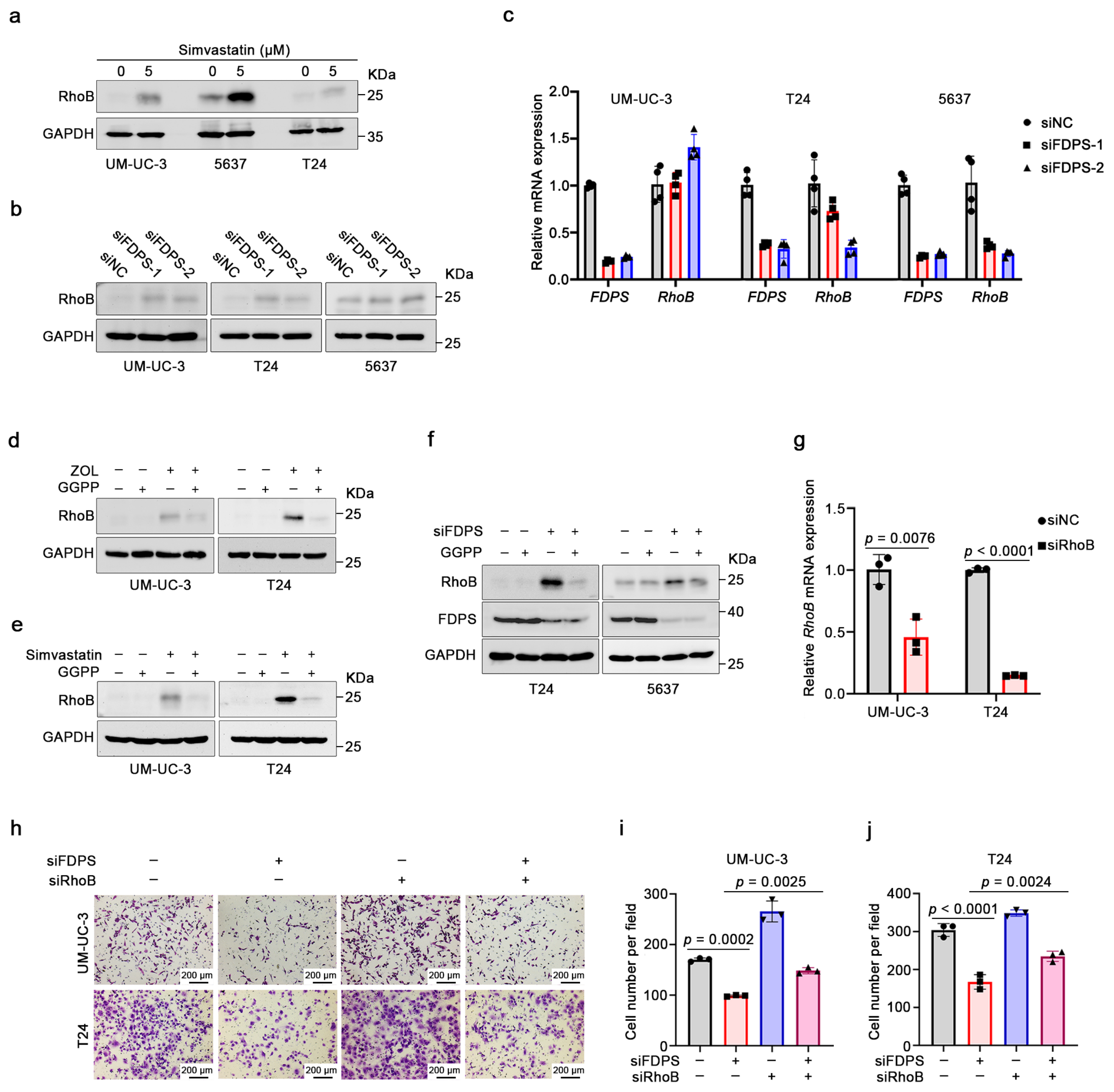

Supplementary Figure 10. RhoB plays an important role in the decreased migration capacity of BLCA cells caused by mevalonate pathway inhibition.

a. RhoB protein expression in simvastatin (0 and 5 μM) treated BLCA cells was detected via Western blotting. **b-c.** The protein and mRNA expression of RhoB in BLCA cells transfected with two different siRNAs against FDPS or with a control siRNA was detected by Western blot (**b**) and qRT-PCR (**c**). **d.** Western blotting was performed to detect RhoB protein expression in BLCA cells after treatment with ZOL and GGPP, respectively, or in combination **e.** Western blotting was performed to detect RhoB protein expression in BLCA cells after treatment with

simvastatin and GGPP, respectively, or in combination. **f.** Western blotting was performed to detect RhoB protein expression in BLCA cells after treatment with siRNAs against FDPS and GGPP, respectively, or in combination. **g.** Validation of the knockdown efficiency of RhoB-specific siRNA by qRT-PCR. **h-j.** Representative images (**h**) and statistical graph (**i** and **j**) of transwell migration assays from the indicated groups after transfection with the described siRNAs for 48 hrs (n = 3). The scale bar is 200 μm. The n number represents n biologically independent experiments in each group. Statistical significance was ascertained by two-tailed unpaired Student's t-test (g) and one-way ANOVA with Dunnett's multiple comparisons test (i and j). The data are shown as the mean ± SD.

Supplementary Figure 11

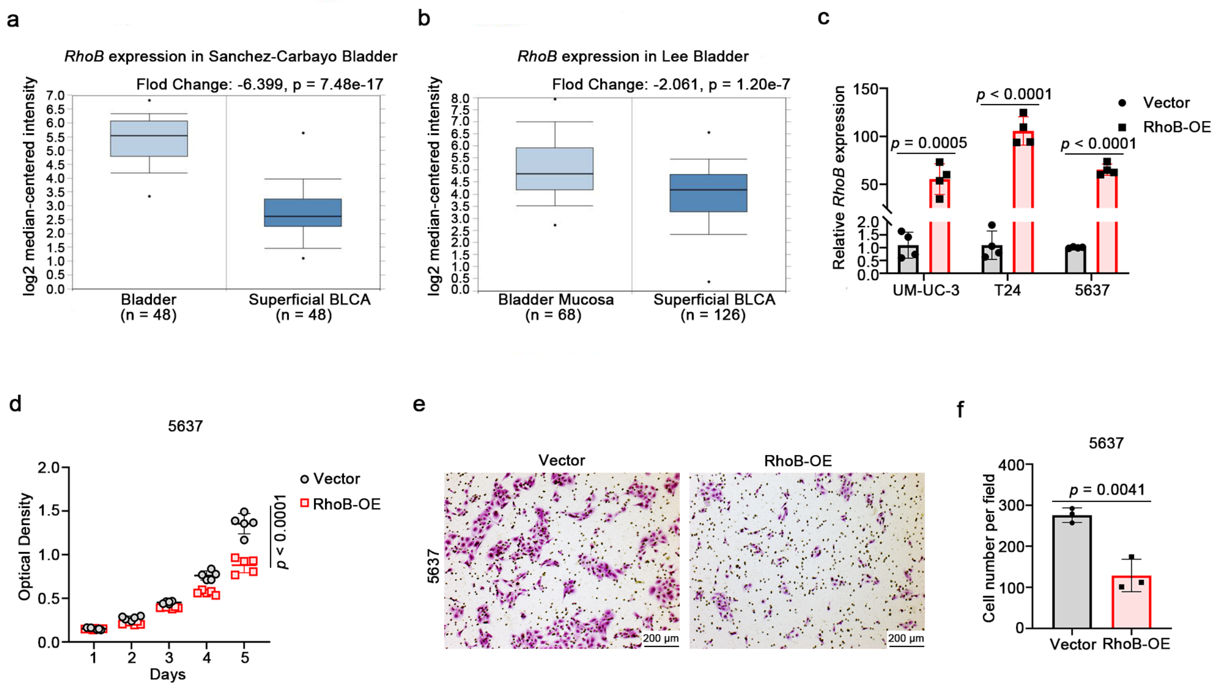

Supplementary Figure 11. RhoB inhibits BLCA metastasis.

a-b. The mRNA level of *RhoB* in BLCA and normal tissues in the Oncomine database. **c.** Validation of the RhoB overexpression efficiency of the RhoB plasmid by qRT-PCR. **d.** MTT assay was performed to detect changes in the proliferation of 5637 cells with or without RhoB overexpression (n = 5). **e-f.** Representative images (**e**) and statistical graph (**f**) of transwell migration assays of 5637 cells transfected with vector or RhoB plasmid (n = 3). The scale bar is 200 μm. The n number represents n biologically independent experiments in each group. Statistical significance was ascertained by two-tailed unpaired Student's t-test (c, d, and f). The data are shown as the mean ± SD.

Supplementary Figure 12

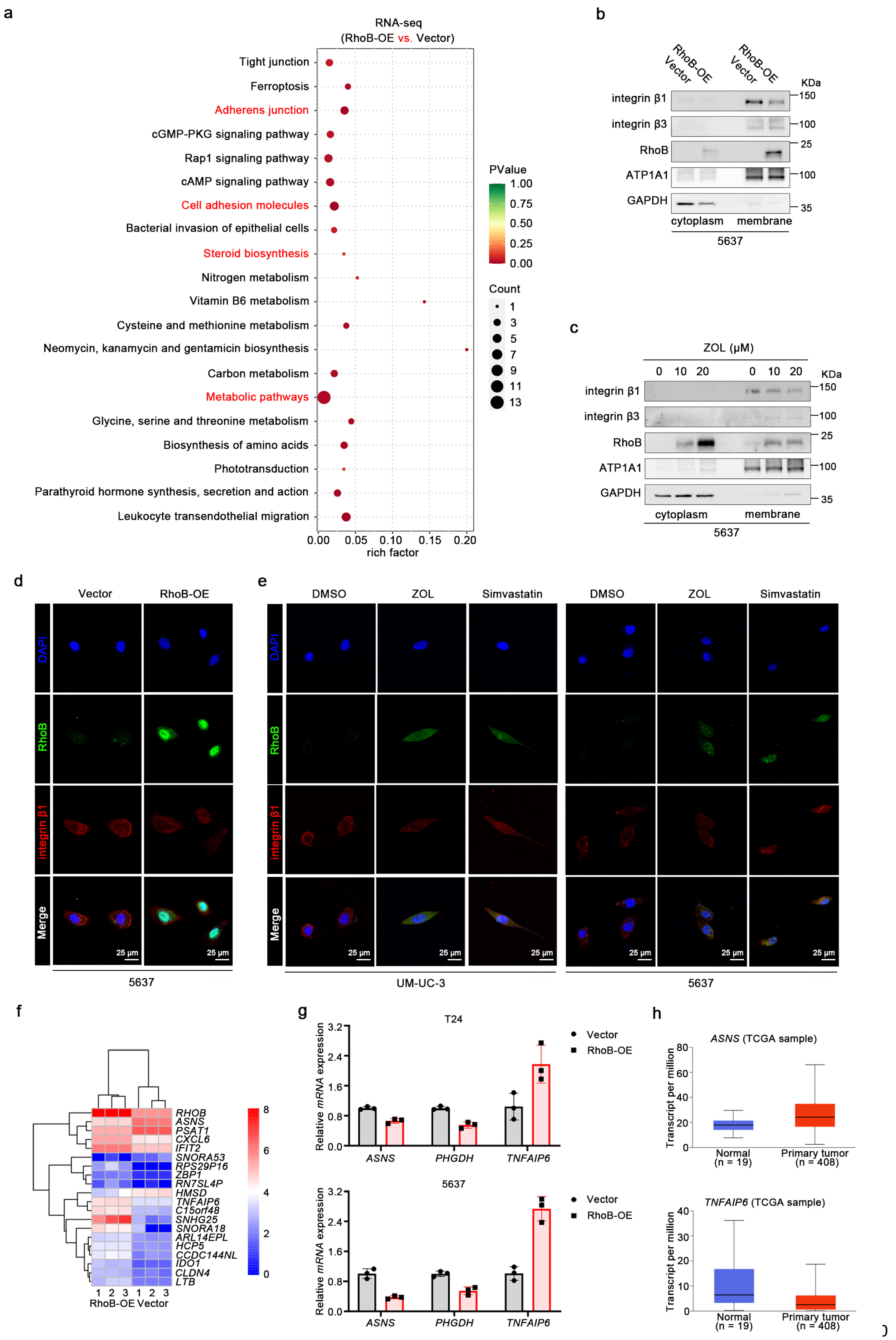

Supplementary Figure 12

Supplementary Figure 12. RhoB is involved in the translocation of integrin β1 from the cytoplasm to the membrane in BLCA cells.

a. Pathway enrichment analysis of DEGs between RhoB overexpression and control T24 cells. **b.** The integrin β1 and β3 proteins in the cytoplasm and on the membrane of 5637 cells transfected with vector or RhoB plasmid were detected via Western blotting. GAPDH and ATP1A1 were used as loading controls for the cytoplasmic and membrane proteins, respectively. **c.** The integrin β1 and β3 proteins in the cytoplasm and membrane of 5637 cells treated with different concentrations of the various concentrations of the various agents were detected via Western blotting. GAPDH and ATP1A1 were used as loading controls for the cytoplasmic and membrane proteins, respectively. **d.** The expression and localization of integrin β1 (red) and RhoB (green) in 5637 cells transfected with the vector or RhoB plasmid were detected via immunofluorescence staining. Nuclei were stained with DAPI (blue). The scale bar is 25 μm. **e.** The expression and localization of integrin β1 (red) or RhoB (green) in UM-UC-3 and 5637 cells treated with ZOL (20 μM) or simvastatin (5 μM) were detected by immunofluorescence staining. Nuclei were stained with DAPI (blue). The scale bar is 25 μm. **f.** Heatmaps of the top 10 significantly upregulated and downregulated genes in RhoB overexpression T24 cells. **g.** The mRNA expression levels of *ASNS*, *PHGDH*, and *TNFAIP6* in RhoB overexpression and control BLCA cells (n = 3). **h.** The mRNA levels of *ASNS* and *TNFAIP6* in BLCA (n = 408) and normal tissues (n = 19) in TCGA-BLCA (RNA-seq data).

Figure 3i

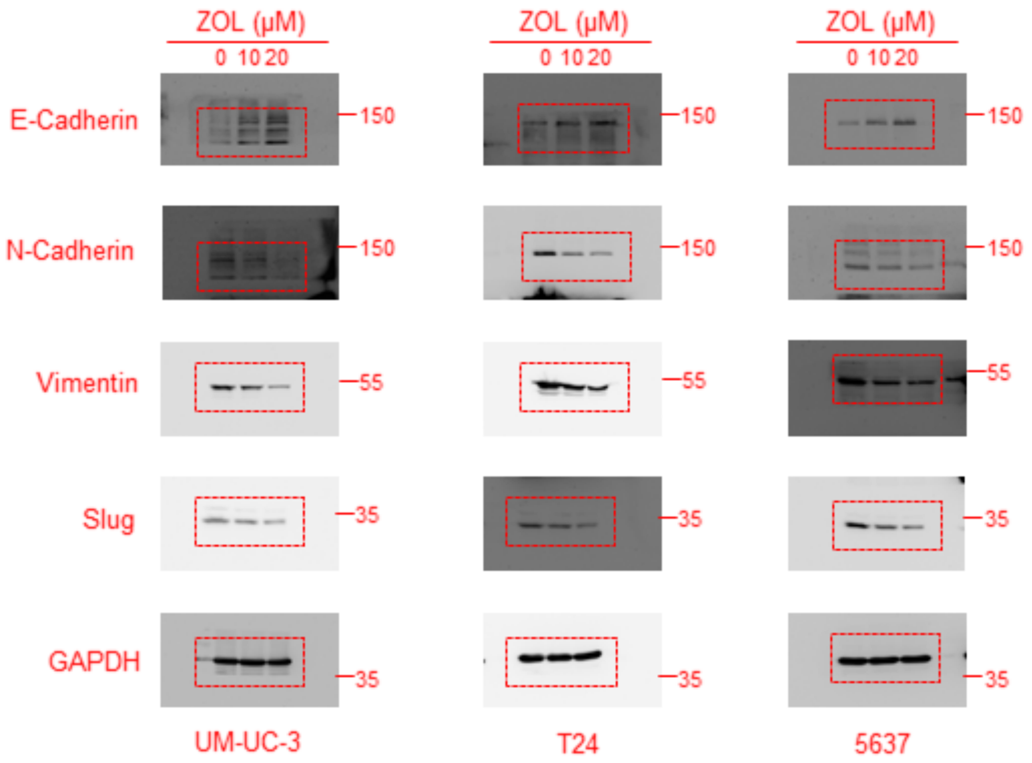

Supplementary Figure 13. Original uncropped Western blots.

Before probing the blots with the indicated antibodies, the membranes were cut to facilitate parallel processing of distinct antigens with their respective antibodies.

Supplementary Figure 13. Original uncropped Western blots.

Figure 4e

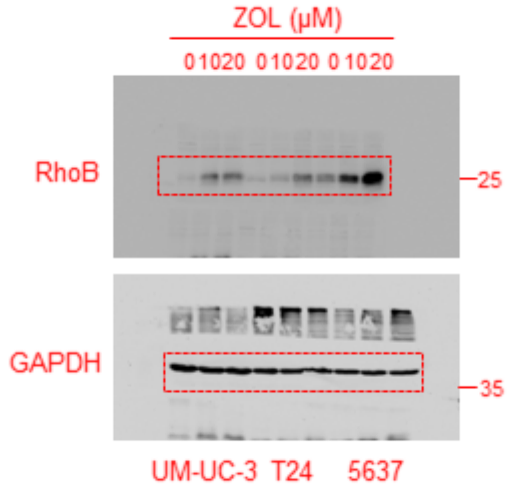

Figure 4k

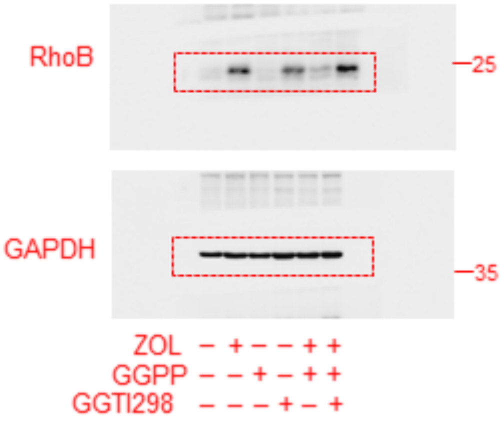

Figure 4l

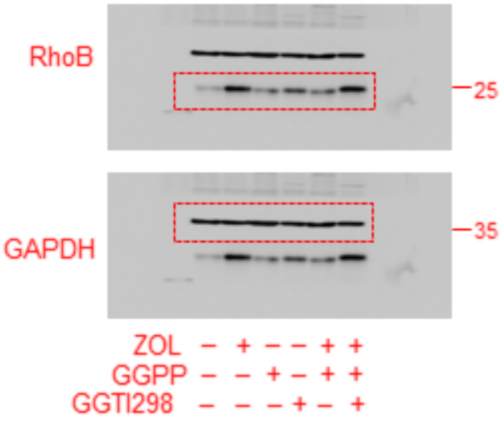

Supplementary Figure 13. Original uncropped Western blots.

Figure 5a

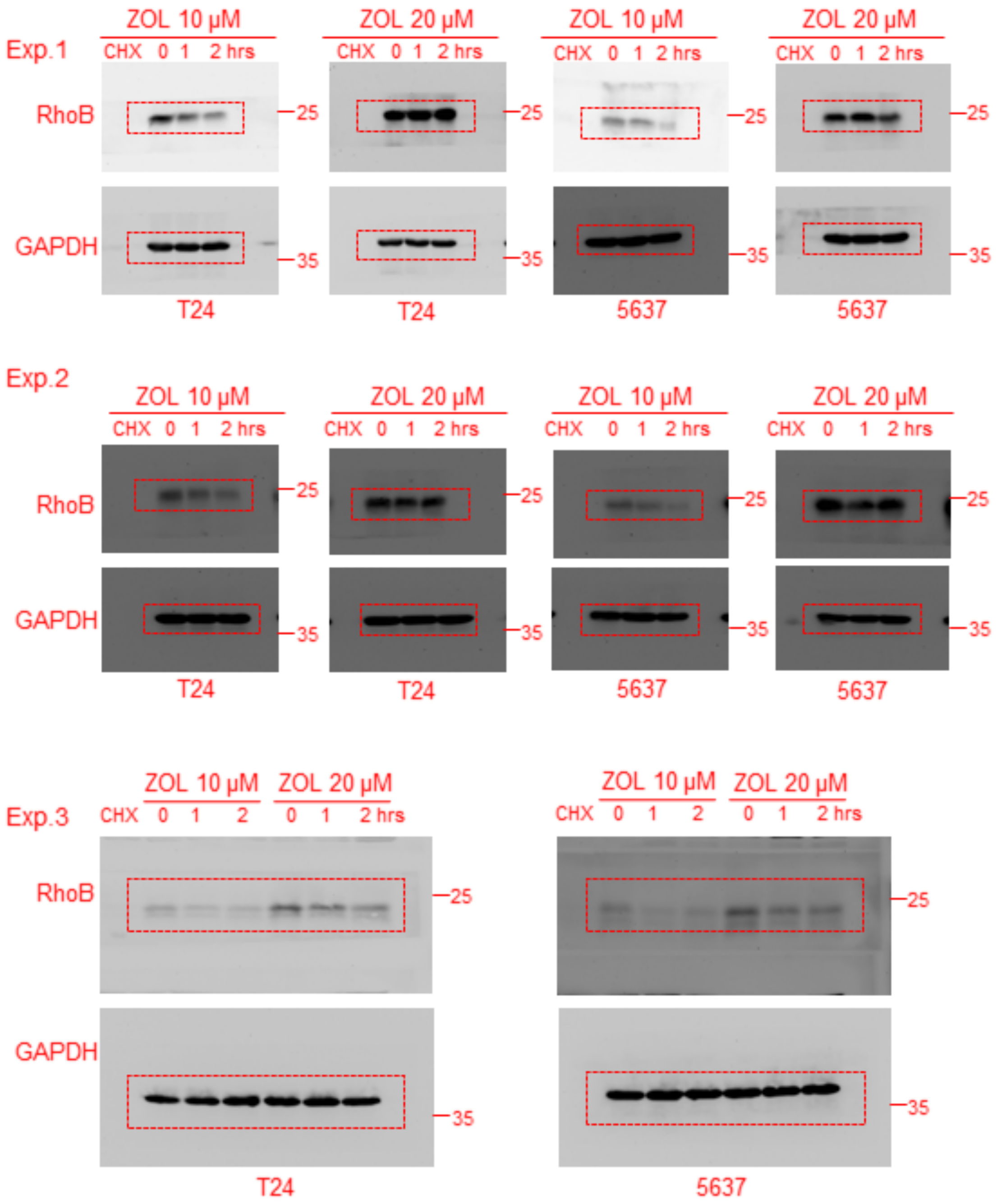

Supplementary Figure 13. Original uncropped Western blots.

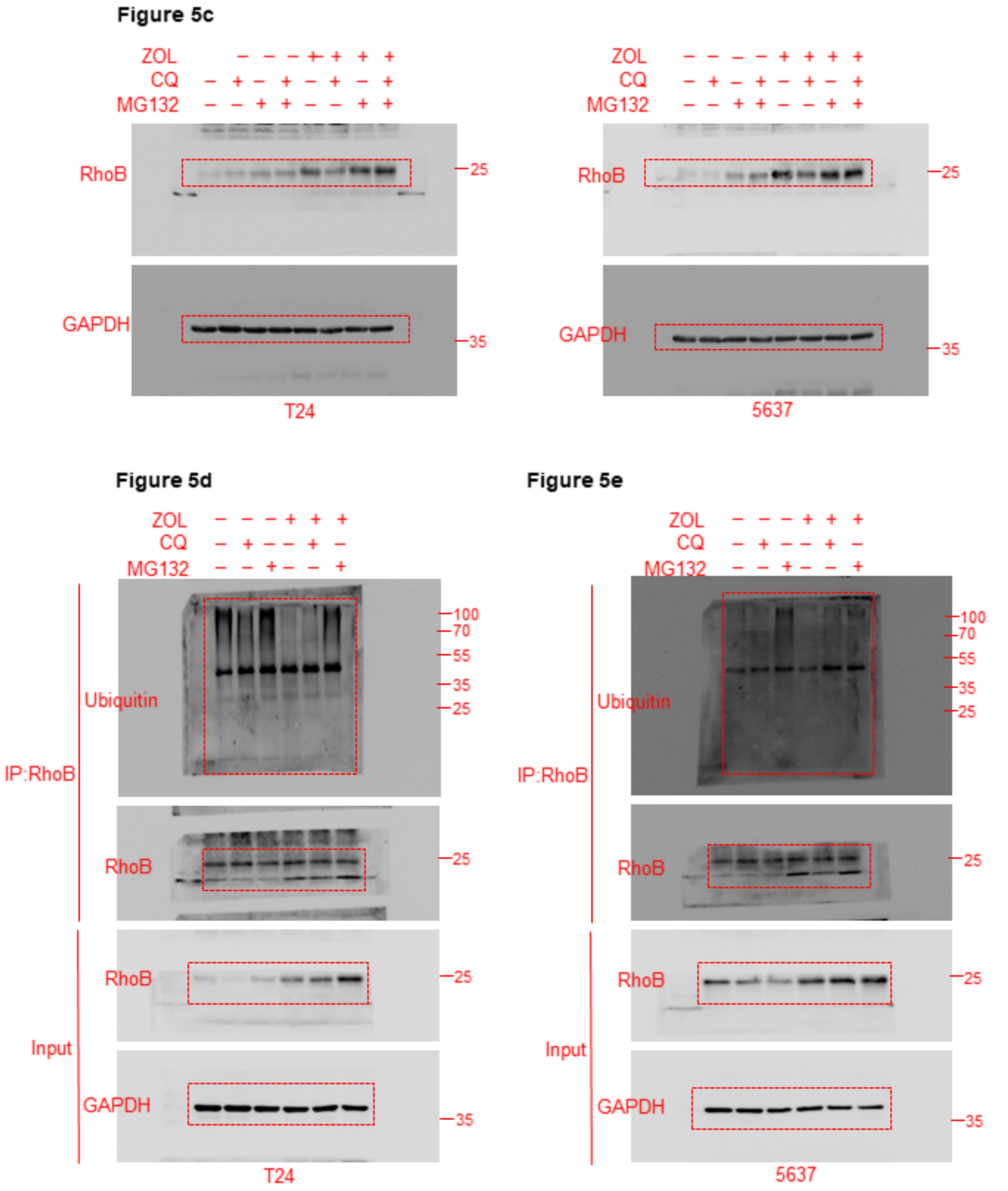

Supplementary Figure 13. Original uncropped Western blots.

Figure 5f

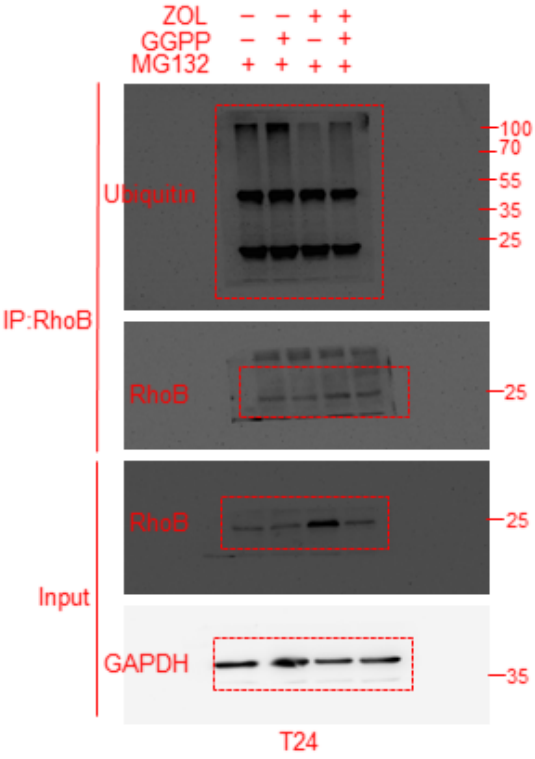

Figure 5g

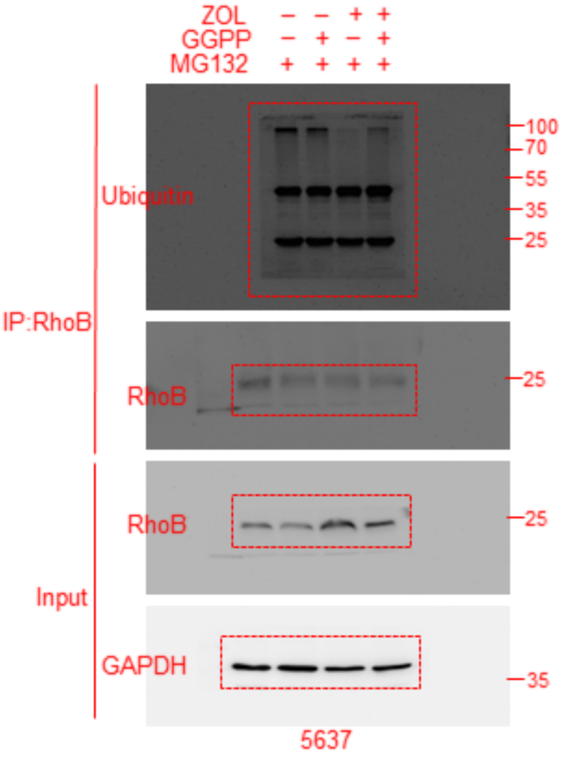

Figure 5h

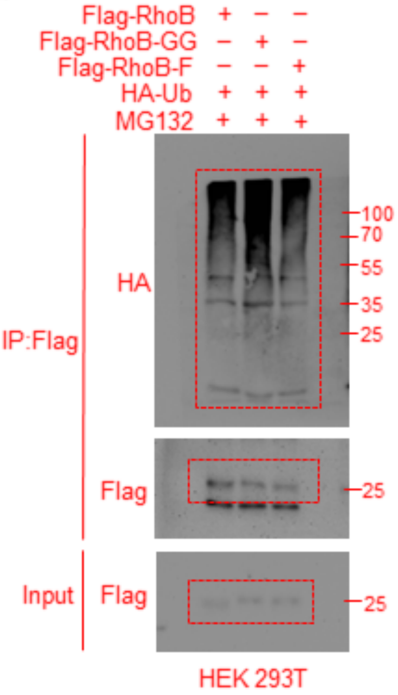

Supplementary Figure 13. Original uncropped Western blots.

Figure 6h

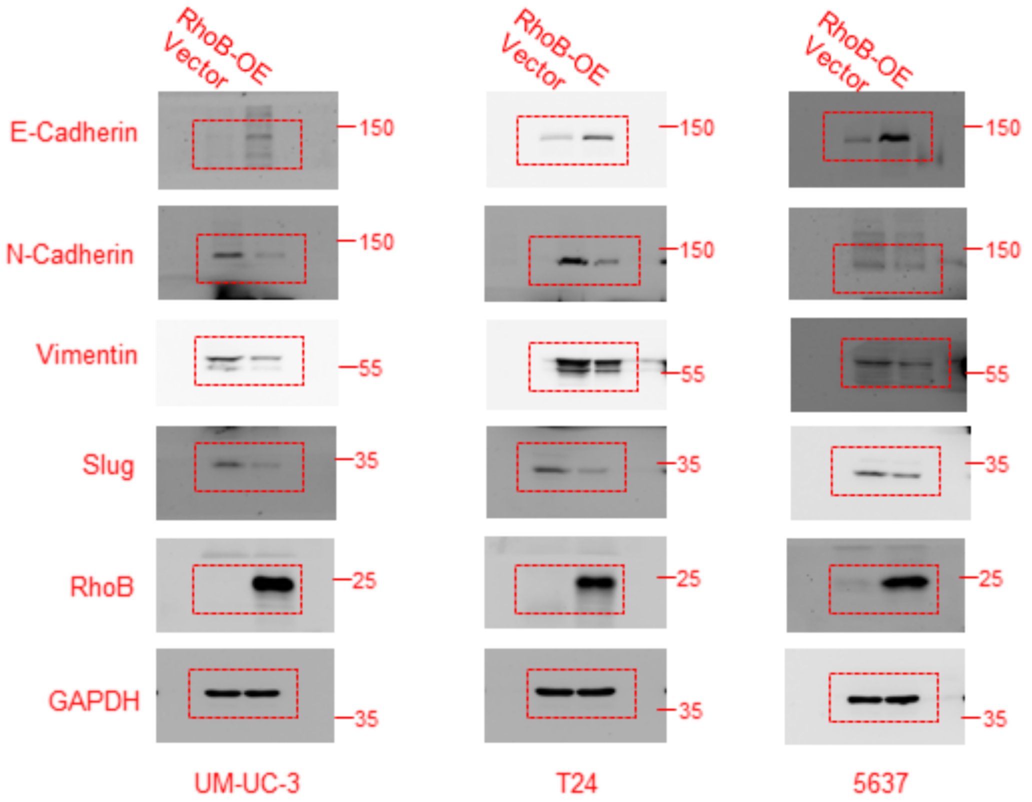

Figure 6i

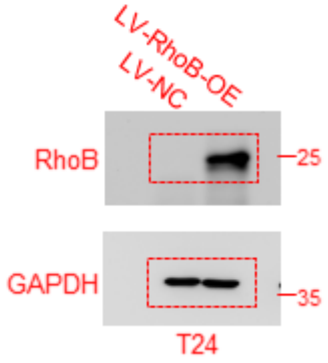

Supplementary Figure 13. Original uncropped Western blots.

Figure 7c

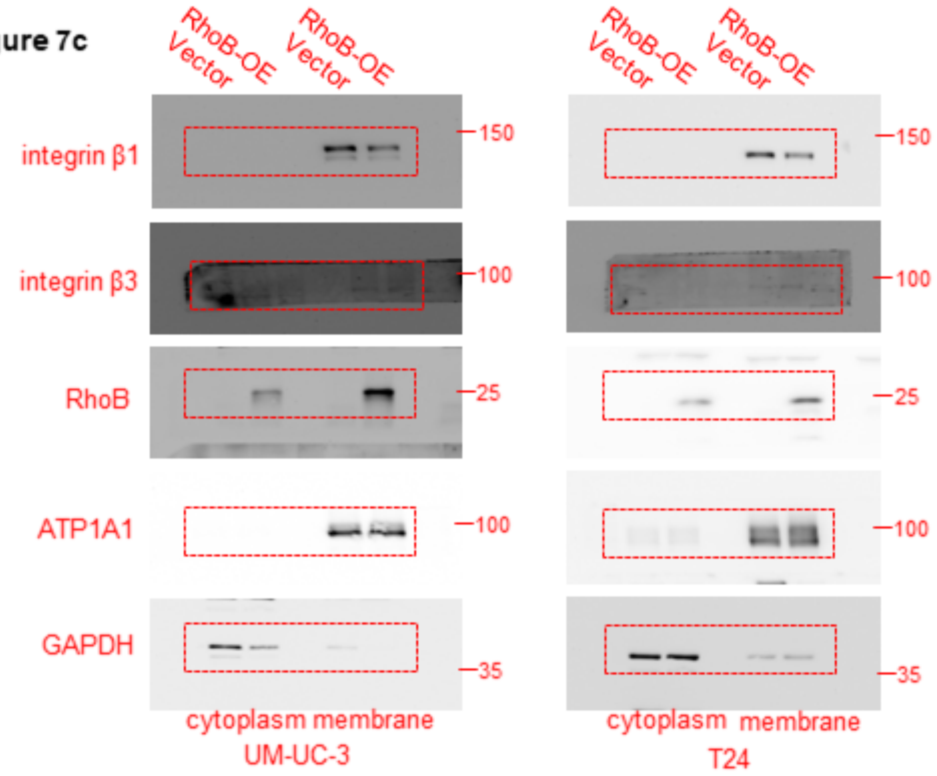

Figure 7d

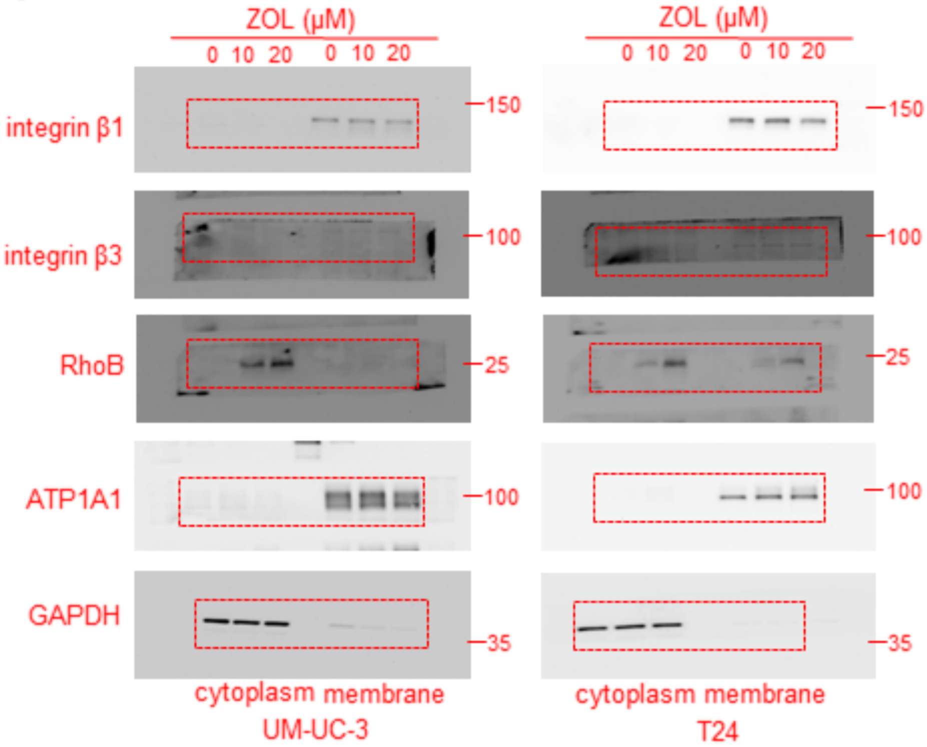

Supplementary Figure 13. Original uncropped Western blots.

Supplementary Figure S3b

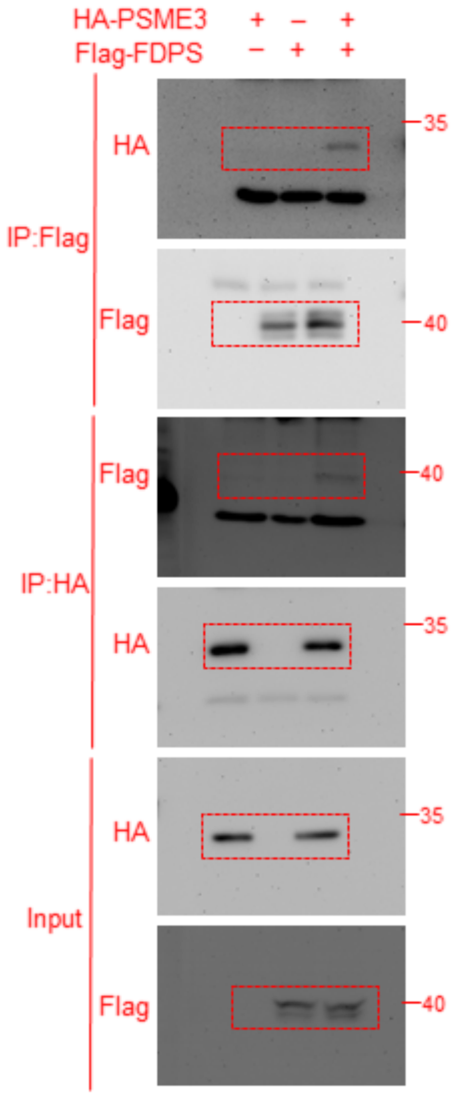

Supplementary Figure S3d

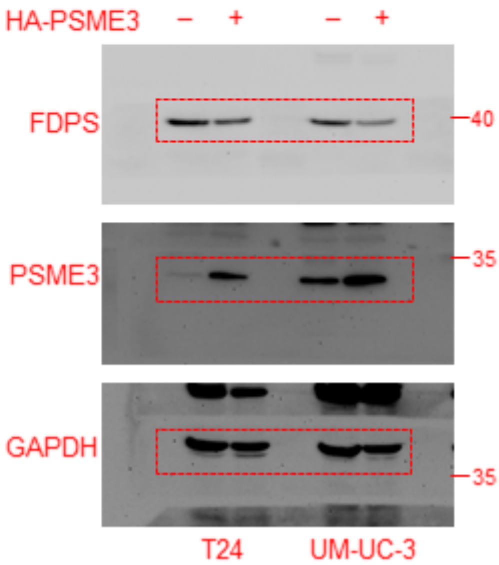

Supplementary Figure S3e

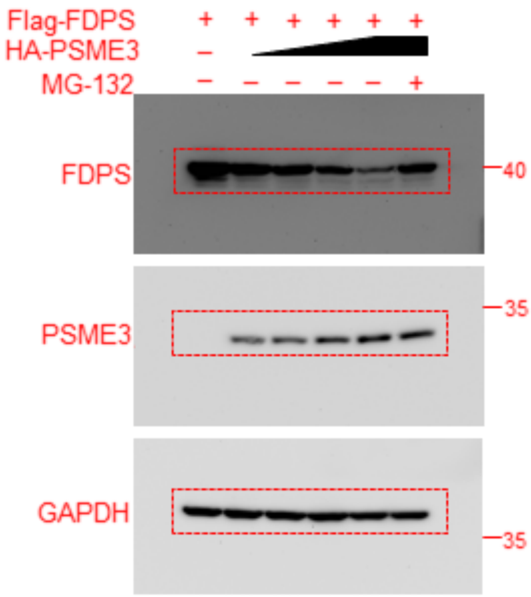

Supplementary Figure 13. Original uncropped Western blots.

Supplementary Figure S3f

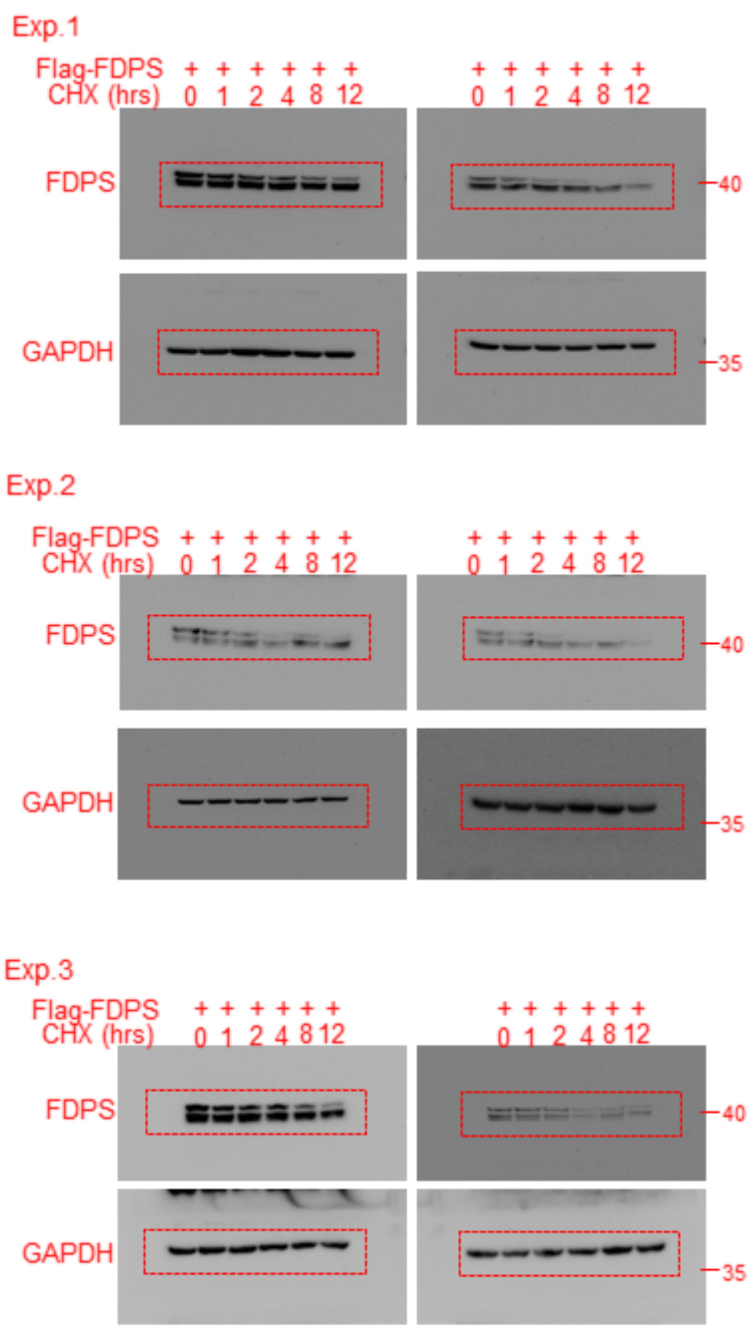

Supplementary Figure S3h

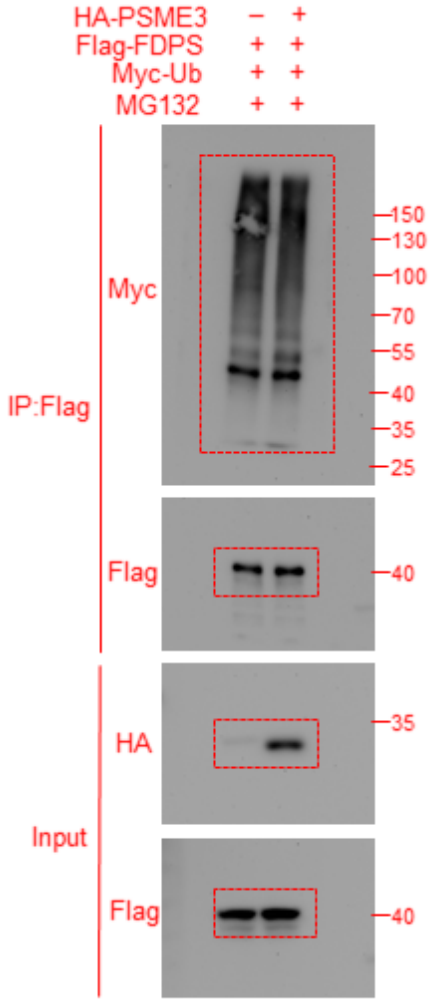

Supplementary Figure 13. Original uncropped Western blots.

Supplementary Figure S4b

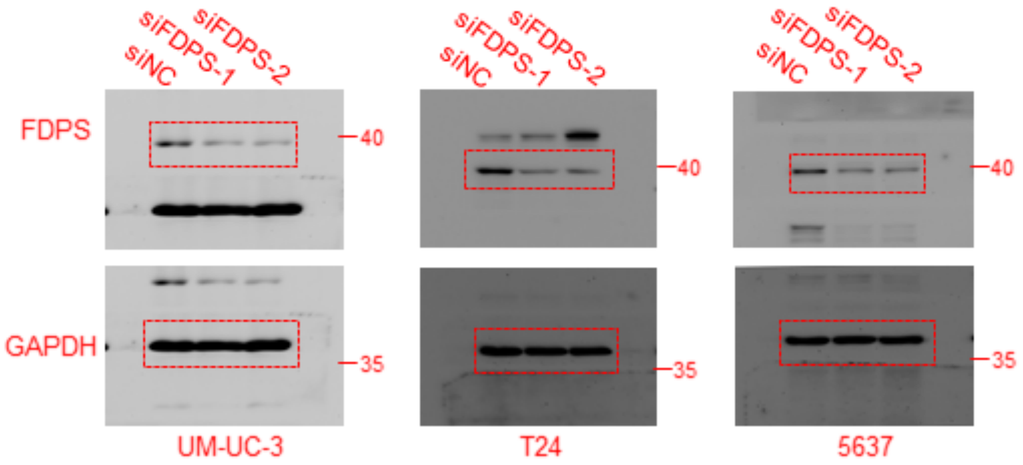

Supplementary Figure S5j

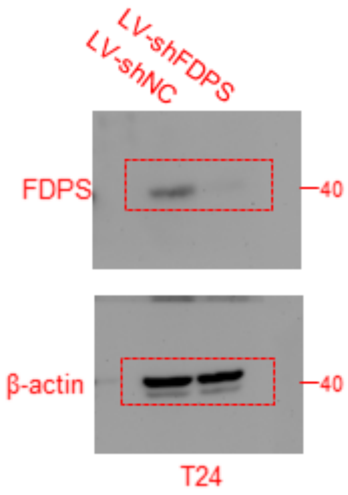

Supplementary Figure 13. Original uncropped Western blots.

Supplementary Figure 10a

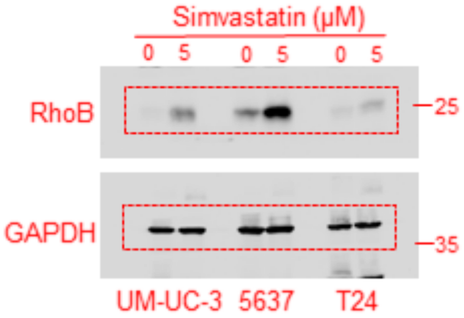

Supplementary Figure 10b

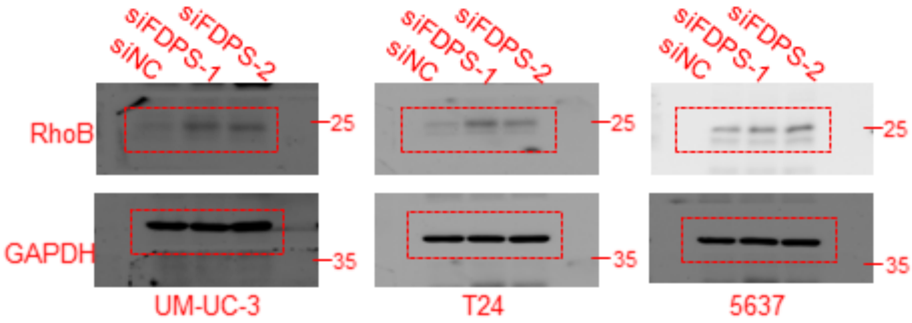

Supplementary Figure 10d

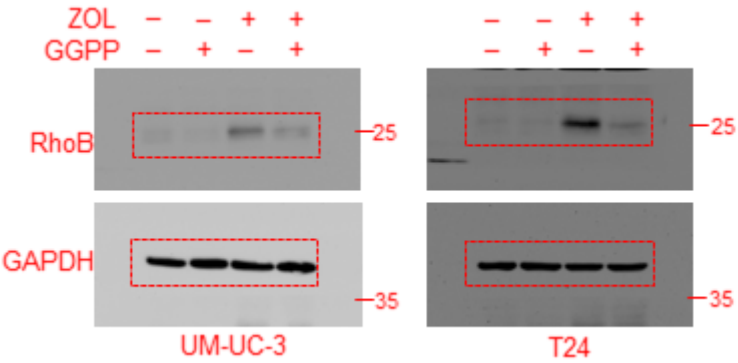

Supplementary Figure 13. Original uncropped Western blots.

Supplementary Figure 10e

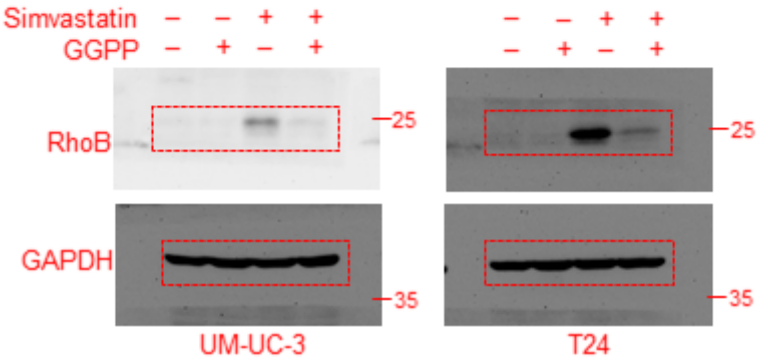

Supplementary Figure 10f

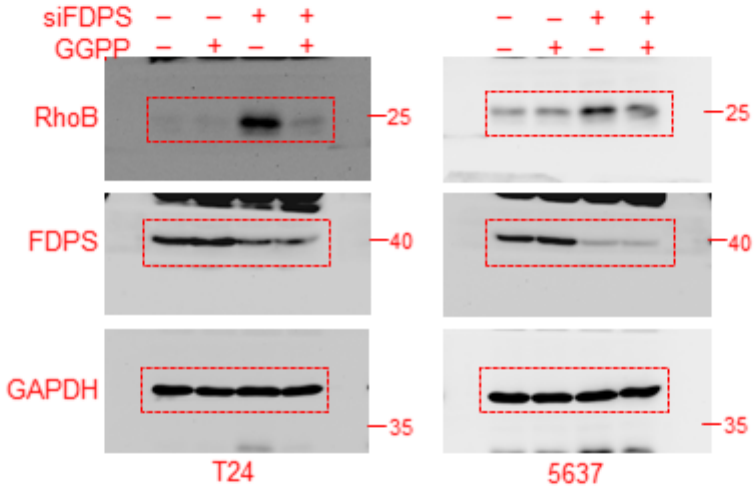

Supplementary Figure 13. Original uncropped Western blots.

Supplementary Figure 12b

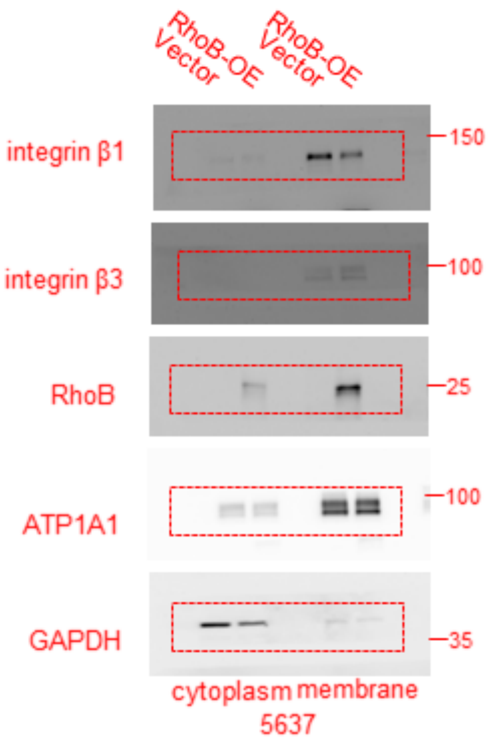

Supplementary Figure 12c

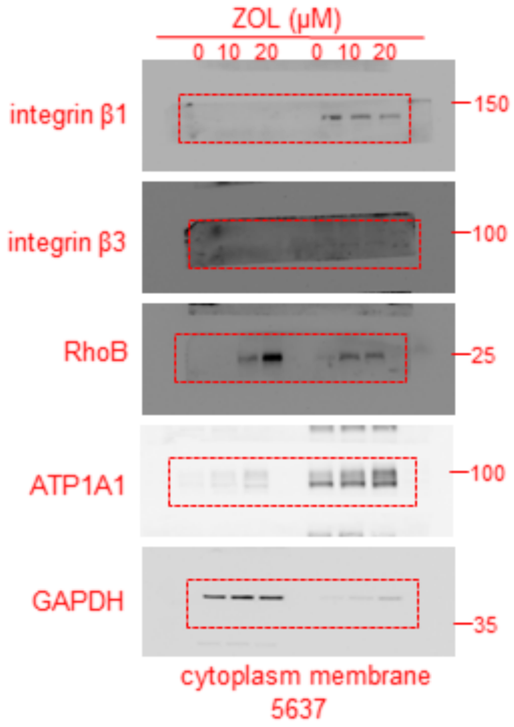

Supplementary Tables 1-4

Supplementary Table 1. Clinicopathological features of BLCA patients in the Zhongnan Hospital cohort.

Patients	Age (year)	Gender	Subtype	Grade	TNM stage
Patient-1	73	Female	MIBC	High	T3aN0Mx
Patient-2	56	Male	MIBC	High	T3N0Mx
Patient-3	66	Male	MIBC	High	T1N0Mx
Patient-4	59	Female	MIBC	High	T4aN0Mx
Patient-5	63	Female	MIBC	High	T2N0Mx
Patient-6	57	Male	MIBC	High	T3aN0Mx
Patient-8	81	Male	MIBC	High	T3aN0Mx
Patient-9	65	Male	MIBC	Low	T1N0Mx
Patient-10	77	Male	MIBC	High	T3N0Mx
Patient-11	62	Male	MIBC	High	T4aN0Mx
Patient-12	79	Male	MIBC	High	T3aN1Mx
Patient-13	82	Male	MIBC	High	T1N0Mx
Patient-14	55	Male	MIBC	High	T1N0Mx
Patient-15	64	Male	MIBC	High	T2N0Mx

MIBC: muscle invasive bladder cancer.

Supplementary Table 2. The siRNA sequences.

Gene	Sense (5’-3’)
siFDPS-1	GCAGAAGGAGGCUGAGAAAUU
siFDPS-2	CCAGCAGUGUUCUUGCAAUTT
siRhoB	ACGUCAUUCUCAUGUGCUUTT
siNC	UUCUCCGAACGUGUCACGUTT

Supplementary Tables

Supplementary Table 3. The primer sequences.

Gene	Forward primer (5'-3')	Reverse primer (5'-3')
GAPDH	GGAGCGAGATCCCTCCAAAAT	GGCTGTTGTCATACTTCCTCATGG
FDPS	TGTGACCGGCAAAATTGGC	GCCCGTTGCAGACACTGAA
RHOB	CTGCTGATCGTGTTCAGTAAGG	TCAATGTCGGCCACATAGTTC
LDLR	TCTGCAACATGGCTAGAGACT	TCCAAGCATTCGTTGGTCCC
SCARB1	AATAAGCCCATGACCCTGAAGC	GCCCCACATGATCTCACCC
NPC1L1	AGAGTGAGCCTTACACAACCA	GCAGGACACGTTGGAGAGT
ABCA1	ACCCACCCTATGAACAACATGA	GAGTCGGGTAACGGAAACAGG
ABCG1	ATTCAGGGACCTTTCCTATTCGG	CTCACCACTATTGAACTTCCCG
TNFAIP6	TTTCTCTTGCTATGGGAAGACAC	GAGCTTGTATTTGCCAGACCG
ASNS	GGAAGACAGCCCCGATTTACT	AGCACGAACTGTTGTAATGTCA
PHGDH	CTGCGGAAAGTGCTCATCAGT	TGGCAGAGCGAACAATAAGGC

Supplementary Tables

Supplementary Table 4. Primary and secondary antibodies.

Antigen	Species source	Dilution	Supplier
E-cadherin	Rabbit	WB/1:500	Cell Signaling Technology, USA, Cat. #3195
N-cadherin	Rabbit	WB/1:500	Cell Signaling Technology, USA, Cat. #13116
Vimentin	Rabbit	WB/1:1000	Cell Signaling Technology, USA, Cat. #5741
Slug	Rabbit	WB/1:1000	Cell Signaling Technology, USA, Cat. #9585
GAPDH	Mouse	WB/1:2000	Santa Cruz, USA, Cat. #sc-365062
RhoB	Rabbit	WB/1:1000; IF/1:100; IP/1μg	Proteintech, China, Cat. #14326-1-AP
FDPS	Rabbit	WB/1:1000	Proteintech, China, Cat. #16129-1-AP
FDPS	Rabbit	IHC/1:200	Abcam, USA, Cat. #ab153805
PSME3	Rabbit	WB/1:1000	Proteintech, China, Cat. #14907-1-AP
β-actin	Mouse	WB/1:2000	Santa Cruz, USA, Cat. #sc-47778
ATP1A1	Rabbit	WB/1:1000	Proteintech, China, Cat. #14418-1-AP
integrin β1	Rabbit	WB/1:1000	Proteintech, China, Cat. #12594-1-AP
integrin β1	Mouse	IF/1:100	Novus, USA, Cat. # MAB17781-SP
integrin β3	Rabbit	WB/1:500	Proteintech, China, Cat. #18309-1-AP
Ubiquitin	Mouse	WB/1:1000	Abcam, USA, Cat. #ab7254
Flag-tag	Mouse	IP/1μg	Sigma, USA, Cat. #F1804
HA-tag	Mouse	IP/1μg; IF/1:100	OriGene, China, Cat. #TA180128
Flag-tag	Rabbit	IF/1:100	Proteintech, China, Cat. #20543-1-AP
Myc-tag	Mouse	IP/1μg	ABclonal, China, Cat. #AE010

WB: Western blot;
IF: Immunofluorescence;
IP: Immunoprecipitation;
IHC: Immunohistochemistry.
