# Supplementary material for: Mevalonate pathway inhibition reduces bladder cancer metastasis by modulating RhoB protein stability and integrin β1 localization
Source: Commun Biol. 2024 Nov 9;7:1476. doi: 10.1038/s42003-024-07067-8 (PMC11550803; doi:10.1038/s42003-024-07067-8)
Supplement: Supplementary file 2 — Description of Additional Supplementary Files [file 42003_2024_7067_MOESM2_ESM.pdf]

Description of Additional Supplementary Files

File name: Supplementary Data 1. IP-MS identification of FDPS-associated proteins.

Description: The data are as follows: (Sheet 1) Results of mass spectrometry of the immunoprecipitation product after IP-Flag in Flag-vector 293T cells. (Sheet 2) Results of mass spectrometry of the immunoprecipitation after IP-Flag in Flag-FDPS-overexpressing 293T cells. (Sheet 3) FDPS-associated proteins are the proteins that are significantly different in the IP-Flag product from Flag-FDPS-overexpressing 293T cells compared to those from Flag-vector 293T cells.

File name: Supplementary Data 2. Source data.

Description: The source data behind the graphs in the paper.
