# Supplementary material for: Mevalonate pathway inhibition reduces bladder cancer metastasis by modulating RhoB protein stability and integrin β1 localization
Source: Commun Biol. 2024 Nov 9;7:1476. doi: 10.1038/s42003-024-07067-8 (PMC11550803; doi:10.1038/s42003-024-07067-8)
Supplement: Supplementary file 5 — Reporting Summary [file 42003_2024_7067_MOESM5_ESM.pdf]

nature portfolio

Corresponding author(s): Yu Xiao, Xinghuan Wang

Last updated by author(s): Sep 9, 2024

Reporting Summary

Nature Portfolio wishes to improve the reproducibility of the work that we publish. This form provides structure for consistency and transparency in reporting. For further information on Nature Portfolio policies, see our Editorial Policies and the Editorial Policy Checklist.

Statistics

For all statistical analyses, confirm that the following items are present in the figure legend, table legend, main text, or Methods section.

n/a	Confirmed	
☐	☒	The exact sample size (*n*) for each experimental group/condition, given as a discrete number and unit of measurement
☐	☒	A statement on whether measurements were taken from distinct samples or whether the same sample was measured repeatedly
☐	☒	The statistical test(s) used AND whether they are one- or two-sided<br>*Only common tests should be described solely by name; describe more complex techniques in the Methods section.*
☒	☐	A description of all covariates tested
☐	☒	A description of any assumptions or corrections, such as tests of normality and adjustment for multiple comparisons
☐	☒	A full description of the statistical parameters including central tendency (e.g. means) or other basic estimates (e.g. regression coefficient) AND variation (e.g. standard deviation) or associated estimates of uncertainty (e.g. confidence intervals)
☐	☒	For null hypothesis testing, the test statistic (e.g. *F*, *t*, *r*) with confidence intervals, effect sizes, degrees of freedom and *P* value noted<br>*Give P values as exact values whenever suitable.*
☒	☐	For Bayesian analysis, information on the choice of priors and Markov chain Monte Carlo settings
☒	☐	For hierarchical and complex designs, identification of the appropriate level for tests and full reporting of outcomes
☒	☐	Estimates of effect sizes (e.g. Cohen's *d*, Pearson's *r*), indicating how they were calculated

Our web collection on statistics for biologists contains articles on many of the points above.

Software and code

Policy information about availability of computer code

Data collection	The qRT-PCR data: iQ™ SYBR® Green Supermix (Bio-Rad, China);<br>The IP-MS data: Liquid Chromatography-Mass system (LC-MS) of Orbitrap Exploris 480 (Thermo Fisher Scientific, USA);<br>The Proteomics date: timsTOF Pro (Bruker Daltonics, GER), UltiMate 3000 RSLCnano system (Thermo, USA) ;<br>The RNA-seq data: illumina Novaseq™ 6000 (Bioprofile,Ltd. Shanghai, China);<br>The WB imaging: chemiluminescence and gel imager (ChemiDoc XRS, Bio-Rad, USA);<br>The immunofluorescence imaging: confocal laser microscope (C2+, Nikon, Japan);<br>The animal fluorescence detection experiment: Small animal in-vivo imaging system xtreme BI (Bruker, Germany).
Data analysis	The statistical analyses, statistical graphs and prognostic curves of the study were performed by R or GraphPad Prism software;<br>The WB quantitative analysis were performed by Image J software;<br>The differentially expressed genes of RNA-seq were analyzed by R package “DEseq2”. The R package “clusterProfiler” was used to perform Gene Set Enrichment Analysis (GSEA), and Kyoto Encyclopedia of Genes and Genomes (KEGG) analysis;<br>The Seurat package was used for scRNA-seq data cleaning and integration. The “FindClusters” function of the Seurat package was used to analyze clustering;<br>MPI was calculated using the Single Sample Genome Enrichment Analysis (ssGSEA) package provided by the R package "GSVA".<br>The NanoPhotometer (Cat. #N60, Implen, Germany) to detect the quality and concentration of the extracted RNA.

For manuscripts utilizing custom algorithms or software that are central to the research but not yet described in published literature, software must be made available to editors and reviewers. We strongly encourage code deposition in a community repository (e.g. GitHub). See the Nature Portfolio guidelines for submitting code & software for further information.

Data

Policy information about availability of data

All manuscripts must include a data availability statement. This statement should provide the following information, where applicable:

- Accession codes, unique identifiers, or web links for publicly available datasets
- A description of any restrictions on data availability
- For clinical datasets or third party data, please ensure that the statement adheres to our policy

The mass spectrometry proteomics data have been deposited to the ProteomeXchange Consortium (http://proteomecentral.proteomexchange.org) via the iProX partner repository with the dataset identifier PXD048067. The RNA sequencing data have been deposited into the NCBI GEO database under the accession numbers GSE252007and GSE270394. The results of IP-MS assays generated in this study are provided in Supplementary Dataset 1. The publicly available source data are accessible from their respective publications, and can be found in the GEO database under the accession numbers: GSE13507, GSE32548, GSE190888, GSE3167, GSE40355, and GSE2450. TCGA-BLCA data were obtained from the Xena Browser (https://xenabrowser.net/). The remaining data can be accessed in the article or in Supplementary Information. The Supplementary Information file contains all supplementary figures (Supplementary Figs. 1-12) and the original uncropped Western blots (Supplementary Fig. 13). Source data are provided with this paper.

Human research participants

Policy information about studies involving human research participants and Sex and Gender in Research.

Reporting on sex and gender	Gender or gender-based analyses were not performed as the study focused on basic molecular mechanisms.
Population characteristics	Fifteen patients with BLCA undergoing radical cystectomy at Department of Urology, Zhongnan Hospital of Wuhan University, were selected. The clinicopathological features of BLCA patients were shown in Supplementary Table 1.
Recruitment	The 15 pairs of human bladder cancer tissues and matched paracancerous tissues were obtained from 15 patients with BLCA after radical cystectomy at Zhongnan Hospital of Wuhan University. The inclusion criteria encompassed BLCA patients requiring radical cystectomy, devoid of metabolism-related diseases, and not taking lipid-lowering drugs or N-BPs. The exclusion criteria included patients with BLCA combined with other tumors, metabolic diseases such as hyperlipidemia and diabetes mellitus, those taking lipid-lowering drugs or N-BPs, and those with secondary bladder tumors.
Ethics oversight	The study was approved by the Medical Ethics Committee of Zhongnan Hospital of Wuhan University (approval number: 2020003) and informed consents were obtained from all individuals.

Note that full information on the approval of the study protocol must also be provided in the manuscript.

Field-specific reporting

Please select the one below that is the best fit for your research. If you are not sure, read the appropriate sections before making your selection.

☒ Life sciences ☐ Behavioural & social sciences ☐ Ecological, evolutionary & environmental sciences

For a reference copy of the document with all sections, see nature.com/documents/nr-reporting-summary-flat.pdf

Life sciences study design

All studies must disclose on these points even when the disclosure is negative.

Sample size	Samples size for each experiment is indicated in the figures or corresponding figure legends.
Data exclusions	Patients with missing survival data were excluded in Fig. 2g.
Replication	Each key in vitro finding was replicated with at least two cell lines. For animal studies, we used at least three replicates. All statistics were based on data from at least three independent replicate experiments.
Randomization	The mice were randomly put into separate/groups cages for experiments.
Blinding	RNA-seq and proteomics samples were analyzed by independent investigators blinded to the experimental conditions/treatments. For animal studies, H&E staining of mice samples were performed by technicians who were blinded to the experimental treatments. The expression of FDPS in the bladder tissues from the tissue microarray was blindly quantified by pathologist.

Reporting for specific materials, systems and methods

We require information from authors about some types of materials, experimental systems and methods used in many studies. Here, indicate whether each material, system or method listed is relevant to your study. If you are not sure if a list item applies to your research, read the appropriate section before selecting a response.

Materials & experimental systems

n/a	Involved in the study
☐	☒ Antibodies
☐	☒ Eukaryotic cell lines
☒	☐ Palaeontology and archaeology
☐	☒ Animals and other organisms
☒	☐ Clinical data
☒	☐ Dual use research of concern

Methods

n/a	Involved in the study
☒	☐ ChIP-seq
☒	☐ Flow cytometry
☒	☐ MRI-based neuroimaging

Antibodies

Antibodies used

Target,Catalog No.,Supplier,Application/ Dilution or amount

For Western blot experiment:
E-cadherin, Cat. # 3195, Cell Signaling Technology, WB/1:1000
N-cadherin, Cat. # 13116, Cell Signaling Technology, WB/1:1000
Vimentin, Cat. # 5741, Cell Signaling Technology, WB/1:1000
Slug, Cat. # 9585, Cell Signaling Technology, WB/1:1000
GAPDH, Cat. # sc-365062, Santa Cruz, WB/1:2000
RhoB, Cat. # 14326-1-AP, Proteintech, WB/1:1000 IP/1μg
FDPS, Cat. # 16129-1-AP, Proteintech, WB/1:1000
PSME3, Cat. # 14907-1-AP, Proteintech, WB/1:1000
β-actin, Cat. # sc-47778, Santa Cruz, WB/1:2000
ATP1A1, Cat. # 14418-1-AP, Proteintech, WB/1:1000
integrin β1, Cat. # 12594-1-AP, Proteintech, WB/1:1000
integrin β3, Cat. # 18309-1-AP, Proteintech, WB/1:500
Ubiquitin, Cat. # ab7254, Abcam, WB/1:1000
Flag-Tag, Cat. # F1804, Sigma, IP/1μg WB/1:1000
HA-Tag, Cat. # TA180128, OriGene, IP/1μg WB/1:1000
Myc-Tag, Cat. # AE010, Abclonal, IP/1μg WB/1:1000

For immunohistochemistry staining:
FDPS, Cat. # ab153805, Abcam, IHC/1:200

For immunofluorescence:
RhoB, Cat. # 14326-1-AP, Proteintech, IF/1:100
integrin β1, Cat. # MAB17781-SP, Novus, IF/1:100
HA-tag, Cat. #TA180128, OriGene, IF/1:100
Flag-tag, Cat. #20543-1-AP, Proteintech, IF/1:100

Validation

All antibodies were purchased from commercial companies, and validated by the data sheets of themanufacturer or citations listed below.
The following primary antibodies were used for Western blot experiments:
1) E-cadherin, Cat. # 3195, Cell Signaling Technology, manufacturer validated for Western blot analysis of extracts from various cell lines and in numerous publications (https://www.cellsignal.cn/products/primary-antibodies/e-cadherin-24e10-rabbit-mab/3195);
2) N-cadherin, Cat. # 13116, Cell Signaling Technology, manufacturer validated for Western blot analysis of extracts from A172 and MCF7 cells (https://www.cellsignal.cn/products/primary-antibodies/n-cadherin-d4r1h-xp-rabbit-mab/13116);
3) Vimentin, Cat. # 5741, Cell Signaling Technology, manufacturer validated for Western blot analysis of extracts from various cell lines and in numerous publications (https://www.cellsignal.cn/products/primary-antibodies/vimentin-d21h3-xp-174-rabbit-mab/5741);
4) Slug, Cat. # 9585, Cell Signaling Technology, manufacturer validated for Western blot analysis of extracts from A204, SKMEL5, and NIH/3T3 cells and in numerous publications (https://www.cellsignal.cn/products/primary-antibodies/slug-c19g7-rabbit-mab/9585);
5) GAPDH, Cat. # sc-365062, Santa Cruz, manufacturer validated for Western blot analysis of GAPDH expression in HeLa, Jurkat, MCF7, A-431 and HL-60 whole cell lysates and in numerous publications (https://datasheets.scbt.com/sc-365062.pdf);
6) RhoB, Cat. # 14326-1-AP, Proteintech, manufacturer validated for Western blot analysis of various lysates and in numerous publications (https://www.ptgcn.com/products/RHOB-Antibody-14326-1-AP.htm);
7) FDPS, Cat. # 16129-1-AP, Proteintech, manufacturer validated for Western blot analysis of extracts from HepG2 cells and in numerous publications (https://www.ptgcn.com/products/FDPS-Antibody-16129-1-AP.htm);
8) PSME3, Cat. # 14907-1-AP, Proteintech, manufacturer validated for Western blot analysis of extracts from COLO 320 cells and in numerous publications (https://www.ptgcn.com/products/PSME3-Antibody-14907-1-AP.htm);
9) β-actin, Cat. # sc-47778, Santa Cruz, manufacturer validated for Western blot analysis of β-Actin expression in HeLa, Jurkat, K-562

and A-431 whole cell lysates and in numerous publications (https://datasheets.scbt.com/sc-47778.pdf);
10) ATP1A1, Cat. # 14418-1-AP, Proteintech, manufacturer validated for Western blot analysis of various lysates and in numerous publications (https://www.ptgcn.com/products/ATP1A1-Antibody-14418-1-AP.htm);
11) integrin β1, Cat. # 12594-1-AP, Proteintech, manufacturer validated for Western blot analysis of extracts from HT-1080 cells and in numerous publications (https://www.ptgcn.com/products/ITGB1-Antibody-12594-1-AP.htm);
12) integrin β3, Cat. # 18309-1-AP, Proteintech, manufacturer validated for Western blot analysis of extracts from Human peripheral blood platelets and in numerous publications (https://www.ptgcn.com/products/ITGB3-Antibody-18309-1-AP.htm);
13) Ubiquitin, Cat. # ab7254, Abcam, manufacturer validated for Western blot analysis of extracts from HeLa cells co-transfected with a plasmid expressing a target protein together with Ubi expressing vector and in numerous publications (https://www.abcam.cn/products/primary-antibodies/ubiquitin-antibody-ubi-1-ab7254.html);
14) Flag-Tag, Cat. # F1804, Sigma, manufacturer validated with Western blot analysis of Flag protein in CHO lysis solution (https://www.sigmaaldrich.cn/deepweb/assets/sigmaaldrich/product/documents/144/194/vol6_iss2_antiflag_m2.pdf);
15) HA-Tag, Cat. # TA180128, OriGene, manufacturer validated with Western blot analysis of extracts from HEK293T cells were transfected with HA tagged LGALS3 cDNA for 48 hrs and lysed (https://cdn.origene.com/datasheet/ta180128.pdf);
16) Myc-Tag, Cat. # AE010, Abclonal, manufacturer validated with Western blot analysis of extracts from 293T transfected with Myc-NLK protein (https://abclonal.com.cn/catalog/AE010).

For immunohistochemistry staining:
1) FDPS, Cat. # ab153805, Abcam, manufacturer validated for Immunohistochemical analysis of paraffin-embedded Ca922 xenograft labeling FDPS/FPS (https://www.abcam.cn/products/primary-antibodies/fdpsfps-antibody-ab153805.html).

For immunofluorescence:
1) RhoB, Cat. # 14326-1-AP, Proteintech, validated for immunofluorescence analysis of HeLa cells (https://www.ptgcn.com/products/RHOB-Antibody-14326-1-AP.htm);
2) integrin β1, Cat. # MAB17781-SP, Novus, manufacturer validated for immunofluorescence analysis of immersion fixed human peripheral blood mononuclear cells (PBMCs) (https://www.novusbio.com/products/integrin-beta-1-cd29-antibody-p5d2_mab17781).
3) HA-tag, Cat. #TA180128, OriGene (https://www.origene.com.cn/catalog/antibodies/tag-antibodies/ta180128/ha-mouse-monoclonal-antibody-clone-cb051)
4) Flag-tag, Cat. #20543-1-AP, Proteintech (https://www.ptgcn.com/products/Flag-Tag-Antibody-20543-1-AP.htm#publications)

Eukaryotic cell lines

Policy information about cell lines and Sex and Gender in Research

Cell line source(s)	Human BLCA cell lines T24, 5637, UM-UC-3, and HEK 293T were both obtained from the Chinese Academy of Sciences (Shanghai, China).
Authentication	Authentication was performed by Cell Bank, Chinese Academy of Sciences (Shanghai, China).
Mycoplasma contamination	All cell lines were tested negative for mycoplasma contamination.
Commonly misidentified lines (See ICLAC register)	None.

Animals and other research organisms

Policy information about studies involving animals; ARRIVE guidelines recommended for reporting animal research, and Sex and Gender in Research

Laboratory animals	4-week-old male BALB/c-nude mice were purchased from Beijing Vital River Laboratory Animal Technology Co. Ltd (Beijing, China). All mice were housed under specific-pathogen-free conditions in a controlled environment room (temperature 20-24°C, relative humidity 30-70% and 12-hrs light-dark cycle) and allowed unrestricted access to food and water.
Wild animals	No wild animals were used in this study.
Reporting on sex	The mice used in the study were all male, because the incidence of bladder cancer is much higher in men than in women (PMID: 27370177).
Field-collected samples	No field-collected samples were used in this study.
Ethics oversight	All work with mice was approved by and performed under the Experimental Animal Welfare and Ethics Committee at Zhongnan Hospital of Wuhan University (approval number: ZN2023114).

Note that full information on the approval of the study protocol must also be provided in the manuscript.
